# Fibrotic lung disease inhibits immune responses to staphylococcal pneumonia via impaired neutrophil and macrophage function

**DOI:** 10.1172/jci.insight.152690

**Published:** 2022-02-22

**Authors:** Helen I. Warheit-Niemi, Summer J. Edwards, Shuvasree SenGupta, Carole A. Parent, Xiaofeng Zhou, David N. O’Dwyer, Bethany B. Moore

**Affiliations:** 1 Department of Microbiology and Immunology and; 2 Department of Pharmacology, University of Michigan, Ann Arbor, Michigan, USA.; 3 Division of Pulmonary and Critical Care Medicine, Department of Internal Medicine, University of Michigan Medical School, Ann Arbor, Michigan, USA.

**Keywords:** Immunology, Pulmonology, Bacterial infections, Fibrosis, Neutrophils

## Abstract

Idiopathic pulmonary fibrosis (IPF) is a progressive and fatal disease characterized by collagen deposition within the lung interstitium. Bacterial infection is associated with increased morbidity and more rapid mortality in IPF patient populations, and pathogens such as methicillin-resistant *Staphylococcus aureus* (MRSA) are commonly isolated from the lungs of hospitalized patients with IPF. Despite this, the effects of fibrotic lung injury on critical immune responses to infection remain unknown. In the present study, we show that, like humans with IPF, fibrotic mice infected with MRSA exhibit increased morbidity and mortality compared with uninfected fibrotic mice. We determine that fibrosis conferred a defect in MRSA clearance compared with nonfibrotic mice, resulting from blunted innate immune responses. We show that fibrosis inhibited neutrophil intracellular killing of MRSA through impaired neutrophil elastase release and oxidative radical production. Additionally, we demonstrate that lung macrophages from fibrotic mice have impaired phagocytosis of MRSA. Our study describes potentially novel impairments of antimicrobial responses upon pulmonary fibrosis development, and our findings suggest a possible mechanism for why patients with IPF are at greater risk of morbidity and mortality related to infection.

## Graphical abstract

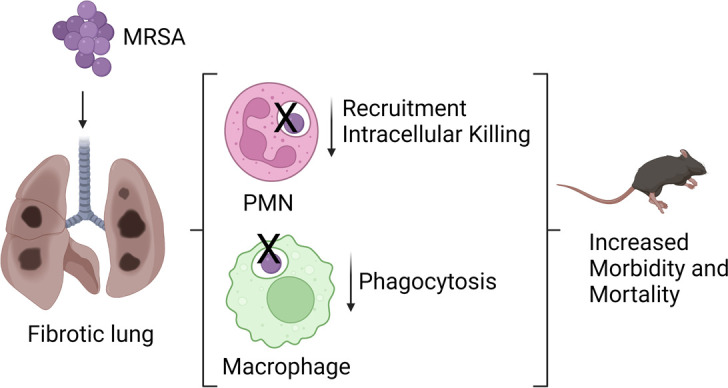

## Introduction

Idiopathic pulmonary fibrosis (IPF) is a devastating and fatal interstitial lung disease (ILD) of unknown cause, characterized by continual progression and poor prognosis (1, 2). The 5-year survival rate of IPF is lower than many cancers, with a median survival time of approximately 3 years following diagnosis (3, 4). The incidence of IPF increases with age, and the prevalence has been reported at 494.5 cases per 100,000 persons aged 65 and older, making IPF one of the most prevalent ILDs (2, 3).

The progression of IPF is thought to result from reoccurring microinjuries to alveolar epithelial cells, in response to which, the lung launches an aberrant wound-healing program resulting in the continued accumulation of myofibroblasts and deposition of collagen (1, 5). Instead of appropriately healing the injured lung tissue, this results in organ fibrosis and eventual respiratory failure (1, 5). While the exact cellular and molecular mechanisms that drive IPF development have not been fully elucidated, considerable progress has been made in this research field; many involved key cell types and signaling molecules have been identified thus far. Notable among these discoveries has been the role of the immune system in disease progression (6). While extensive research has been devoted to understanding how changes in the immune response contribute to further fibrotic development, the effects of these alterations on critical responses to infection are unknown.

Abundant clinical and experimental data indicate that patients with IPF are uniquely susceptible to morbidity and mortality caused by respiratory infections. Several studies examining mortality in patients with IPF identify an infection either as an immediate cause of death or during autopsy (7–11).

There is a clear link between the presence of potentially pathogenic bacteria within the lung and decreased survival in IPF patient populations. It is known that patients with IPF have increased bacterial burdens in bronchoalveolar lavage fluid compared with healthy controls and that baseline bacterial burden predicts the rate of lung function decline in patients with IPF, with higher bacterial burdens correlating with increased risk of death (12, 13). Of interest are some reports demonstrating that treatment of IPF patients with prophylactic antibiotics reduces all-cause mortality and decreases rates of nonelective hospitalization (14, 15). One bacterial species commonly isolated from the lungs of patients with IPF is *Staphylococcus aureus* (*S*. *aureus*), particularly methicillin-resistant *S*. *aureus* (MRSA), and the bacterial genus *Staphylococcus* has been associated with more rapid IPF disease progression (8, 9, 11, 16).

In this study, we use staphylococcal pneumonia caused by MRSA as a clinically relevant model to assess immune responses after bleomycin-induced lung fibrosis. We demonstrate that fibrotic mice infected with MRSA exhibited increased morbidity and mortality compared with mice treated with bleomycin or MRSA alone, mirroring clinical findings in patients with IPF. In addition, we show that fibrotic lung disease induces a global downregulation of antibacterial responses, resulting in impaired bacterial clearance in the lung. This defect corresponded with decreased recruitment of neutrophils and an altered lung inflammatory profile. We also find that neutrophils and macrophages in bleomycin-treated mice exhibited decreased antimicrobial effector functions, showing that fibrotic lung disease impaired the function of innate immune cells. Taken together, this study demonstrates several mechanisms by which immune responses are suppressed in lung fibrosis. The present findings may provide a more complete understanding of why infection worsens prognosis in IPF.

## Results

### Fibrotic mice are more susceptible to morbidity and mortality after MRSA infection.

**Figure 1:**
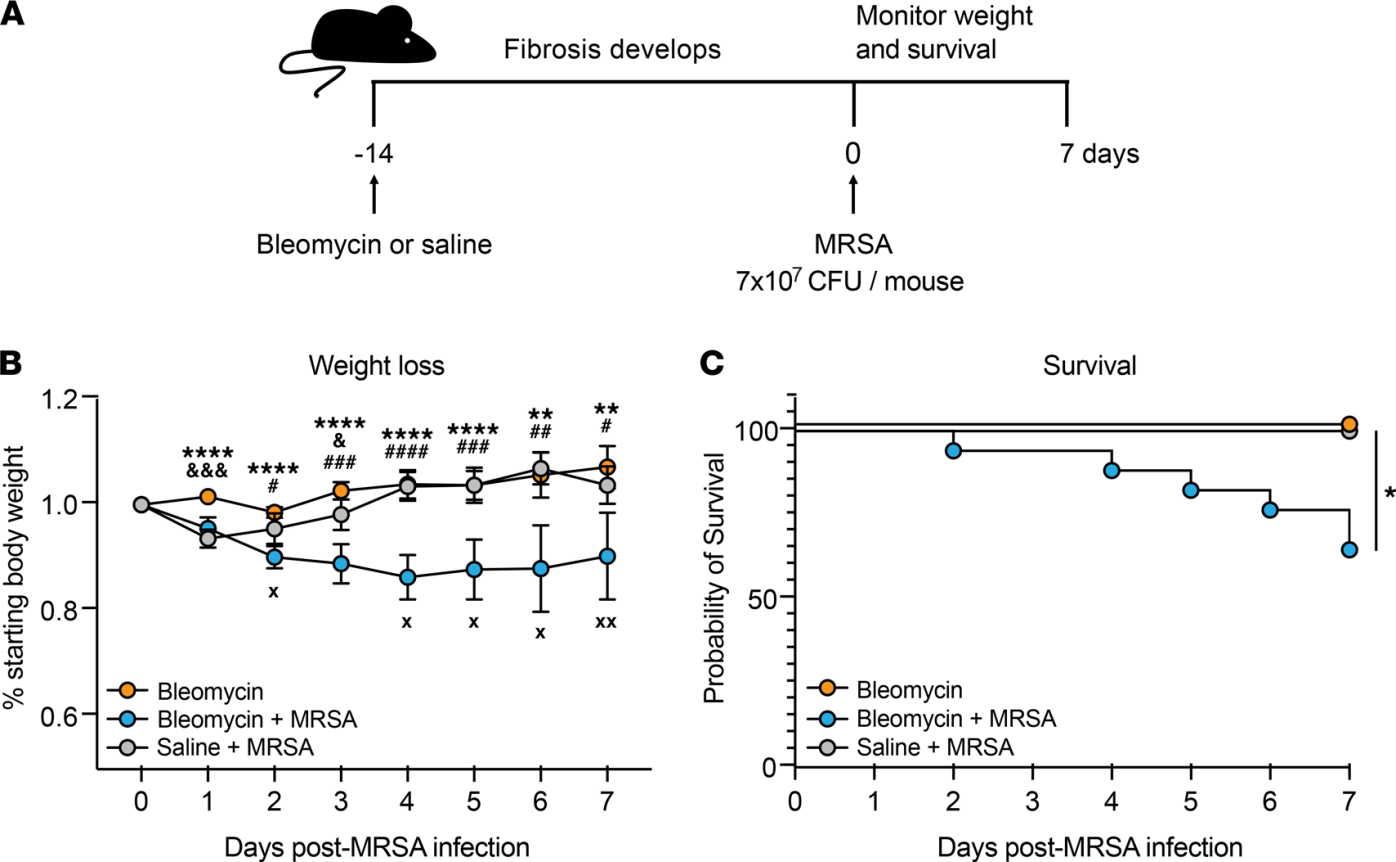
MRSA infection worsens outcome after fibrotic lung injury. (**A**) Mice were treated with bleomycin or saline 14 days prior to infection with MRSA (7 × 10^7^ CFU/mouse). Survival and weight loss were monitored for 7 days following infection. (**B**) Weight loss was monitored as a function of percentage of starting body weight in mice treated with bleomycin (*n* = 6), bleomycin + MRSA (*n* = 9), or saline + MRSA (*n* = 5) as described in **A**. Data from 1 experiment. Bars represent the means ± SD. Statistical analysis by mixed-effects analysis with Tukey’s multiple-comparison test. Statistical comparisons: **P < 0.01, ****P < 0.0001 bleomycin vs. bleomycin + MRSA; ^&^*P* < 0.05, ^&&&^*P* < 0.001 bleomycin vs. saline + MRSA; ^#^*P* < 0.05, ^##^*P* < 0.01, ^###^*P* < 0.001, ^####^*P* < 0.0001 bleomycin + MRSA vs. saline + MRSA. X, death in bleomycin + MRSA group. (**C**) Survival was monitored daily in mice treated with bleomycin (*n* = 14), bleomycin + MRSA (*n* = 17), or saline + MRSA (*n* = 5) as described in **A**. Combined data from 2 independent experiments. Statistical analysis by log-rank test. *P < 0.05.

We developed a murine model of sequential pulmonary fibrosis and infection by utilizing bleomycin-induced fibrosis followed by oropharyngeal inoculation with MRSA, a pathogen that has previously been isolated from patients with IPF (8, 9, 11). To first assess how MRSA pneumonia affects morbidity and mortality as fibrosis develops, we infected mice with 7 × 10^7^ CFU 14 days after bleomycin treatment and monitored weight loss and survival for 7 days compared with mice treated with saline + MRSA and bleomycin alone (Figure 1A). We found that, while infection of both bleomycin + MRSA–treated (fibrotic) and saline + MRSA–treated (nonfibrotic) mice resulted in an initial decrease in body weight, nonfibrotic mice completely recovered within 4 days of infection, with percentage starting body weight returning to the level of mice treated with bleomycin alone (Figure 1B). Mice treated with bleomycin + MRSA continued to lose weight and did not recover within 7 days of infection (Figure 1B and Supplemental Figure 1A; supplemental material available online with this article; https://doi.org/10.1172/jci.insight.152690DS1). This was reflected in survival measurements, as 100% of bleomycin- or MRSA-treated mice survived to day 7 postinfection, while treatment with MRSA following bleomycin resulted in only 65% survival to this time point (Figure 1C). The absolute body weight of mice treated with bleomycin did not significantly differ from the weight of mice treated with saline at the time of infection, showing that this effect was not due to increased morbidity in the fibrotic group prior to infection (Supplemental Figure 1B). Importantly, decreased survival after bleomycin + MRSA treatment was not due to enhanced fibrotic development or increased lung injury compared with bleomycin treatment alone, as demonstrated by measurements of collagen deposition and lung leak, respectively (Supplemental Figure 2, A–C).

### The development of fibrosis impairs MRSA clearance.

**Figure 2:**
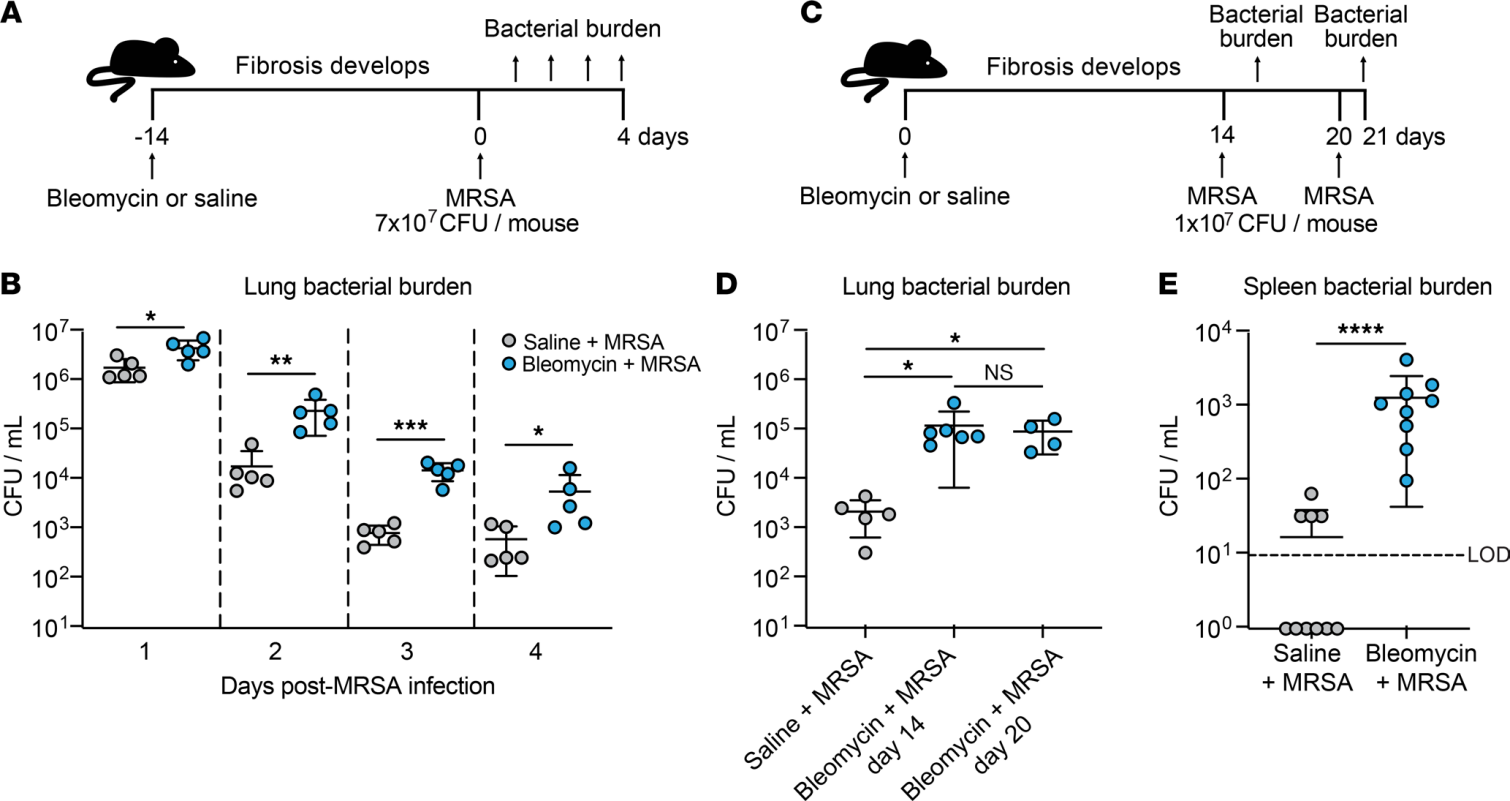
Fibrotic lung disease impairs MRSA clearance 14 and 21 days after bleomycin treatment. (**A**) Mice were treated with bleomycin or saline 14 days prior to infection with MRSA (7 × 10^7^ CFU/mouse). Lung bacterial burden was quantified 1–4 days after infection. (**B**) Lung bacterial burden from mice described in **A**. Bacterial burden was quantified at specified time points (*n* = 5 mice per group). Representative of 2 independent experiments. Data represent the means ± SD. Statistical analysis by multiple Student’s *t* tests. *P < 0.05, **P < 0.01, ***P < 0.001. (**C**) Mice were treated with saline or bleomycin and then infected with 1 × 10^7^ CFU MRSA 14 or 20 days after bleomycin treatment. Lung bacterial burden was quantified 24 hours postinfection (day 15 or day 21). Saline-treated mice were infected on day 20 and MRSA was quantified on day 21. (**D**) Lung bacterial burden from mice described in **C** (saline + MRSA *n* = 5, bleomycin + MRSA day 14 *n* = 6, bleomycin + MRSA day 20 *n* = 4). Representative of 2 independent experiments. Data represent the means ± SD. Statistical analysis by Kruskal-Wallis test with Dunn’s multiple comparisons. *P < 0.05. (**E**) Spleen bacterial burden from mice infected with 1 × 10^7^ CFU MRSA 20 days after bleomycin (*n* = 9) or saline (*n* = 10) treatment. CFU measured 24 hours postinfection. Data from 2 combined independent experiments. Data represent the means ± SD. Statistical analysis by Mann-Whitney *U* test. ****P < 0.0001. LOD, limit of detection.

Given the enhanced morbidity and mortality in bleomycin + MRSA–treated mice and our observation that this did not correlate with enhanced fibrosis, we next sought to investigate how pulmonary fibrosis affects bacterial clearance in fibrotic mice compared with nonfibrotic mice. Mice were infected with 7 × 10^7^ CFU (“high dose”) MRSA 14 days after bleomycin or saline treatment, and bacterial CFU were enumerated 1–4 days after infection (Figure 2A). We found that fibrotic mice exhibited a significant defect in bacterial clearance compared with nonfibrotic mice at all time points measured (Figure 2B). Because we observed significant mortality in mice infected with our “high dose” of MRSA (as described in Figure 1C), we next compared bacterial burden in saline-treated mice or mice infected on day 14 or 20 after bleomycin with 1 × 10^7^ CFU (“low dose”) and harvested 24 hours postinfection (Figure 2C). Because day 14 after bleomycin treatment is considered early-stage fibrosis in this model, we wanted to verify that the defect in bacterial clearance persisted into late-stage fibrosis (17). We found that mice infected at day 14 or day 20 after bleomycin treatment had a similar bacterial clearance defect and similarly elevated lung hydroxyproline levels compared with nonfibrotic infected mice (Figure 2D and Supplemental Figure 3). Because the half-life of bleomycin is approximately 10 minutes in susceptible mice, these results, seen 2–3 weeks later, cannot be attributed to direct antimicrobial effects of the drug (18). We also assessed MRSA dissemination out of the lung by quantifying bacterial burden in spleens from bleomycin- and saline-treated mice and measured significantly increased MRSA CFU in fibrotic mice compared with nonfibrotic mice (Figure 2E).

### Fibrotic lung disease induces a broad downregulation in immune responses to bacterial infection.

**Figure 3:**
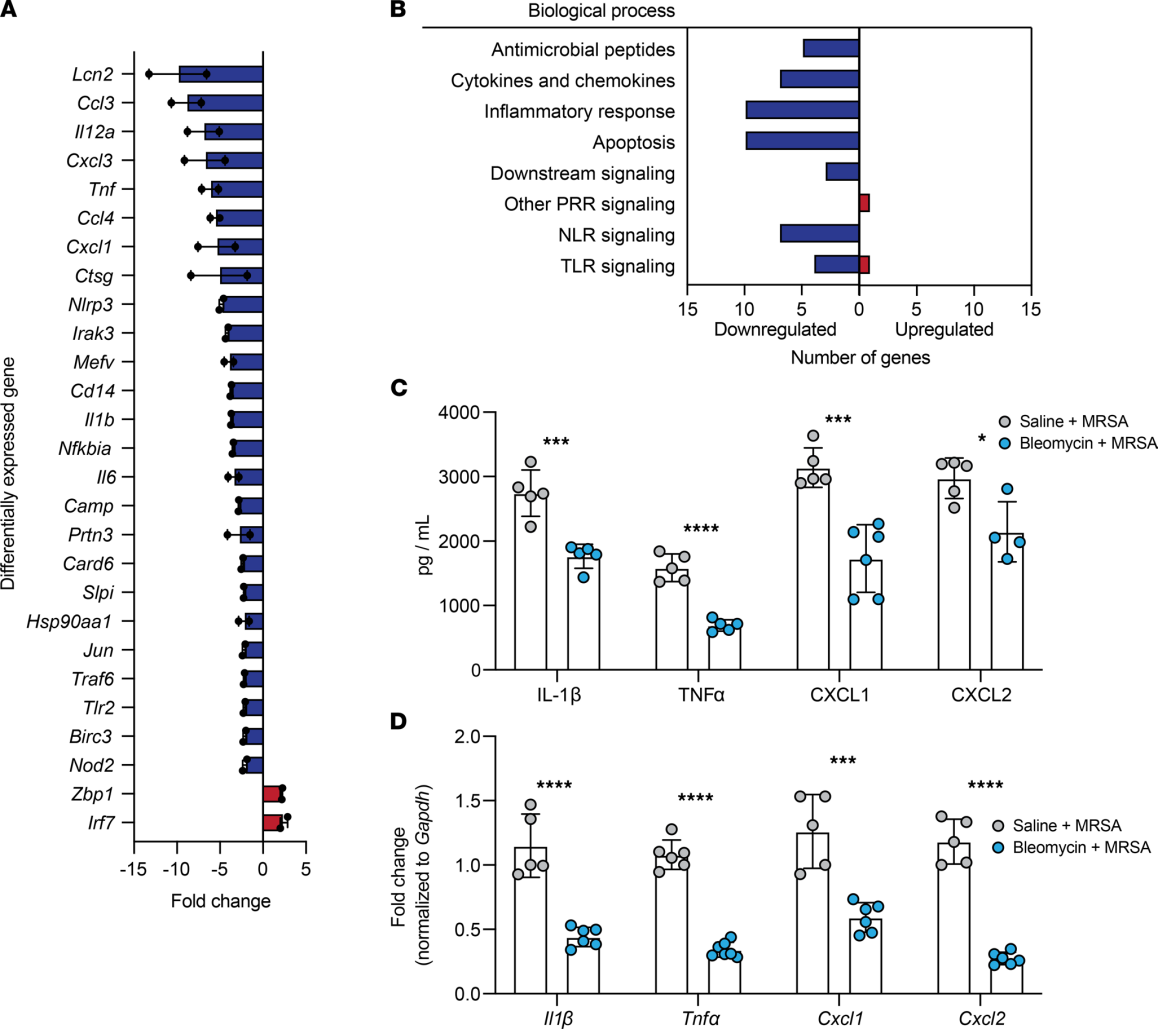
Fibrotic lung disease inhibits antibacterial immune responses after MRSA infection 21 days after bleomycin. (**A**) Antibacterial response PCR array (QIAGEN) was used to measure gene expression of total lung cells from mice 21 days after treatment with bleomycin (*n* = 4) or saline (*n* = 5), isolated 24 hours after infection with MRSA (1 × 10^7^ CFU). Differentially expressed genes shown as fold change of bleomycin + MRSA samples relative to saline + MRSA. Results are the average of 2 independent experiments. Samples from each experiment represent combined biological replicates. (**B**) Numbers of genes, as identified in **A**, in bleomycin + MRSA samples corresponding to different antimicrobial processes that were up- or downregulated relative to saline + MRSA samples. (**C**) IL-1β, TNF-α, CXCL1, and CXCL2 measured via ELISA of whole lung homogenate from mice treated with bleomycin + MRSA (*n* = 4–6) or saline + MRSA (*n* = 5). Representative of 2 independent experiments. Data represent the means ± SD. Statistical analysis by unpaired Student’s *t* test. *P < 0.05, ***P < 0.001, ****P < 0.0001. (**D**) *Il1*β, *Tnf*α, *Cxcl1*, and *Cxcl2* measured via RT-qPCR of total lung cells isolated from mice treated with bleomycin + MRSA (*n* = 6–7) or saline + MRSA (*n* = 5). Representative of 2 independent experiments. Data represent the means ± SD. Statistical analysis by unpaired Student’s *t* test. ***P < 0.001, ****P < 0.0001.

To determine if impaired immune responses to infection played a role in the increased morbidity, mortality, and bacterial burden observed after bleomycin + MRSA treatment, we utilized RT^2^ Profiler PCR arrays (QIAGEN) to broadly assess the expression of genes involved in antibacterial responses in total lung cells isolated from mice 21 days after treatment with bleomycin + MRSA or saline + MRSA. Out of 79 genes quantified, 27 were differentially expressed in fibrotic infected mice (defined as up- or downregulation greater than 2-fold compared with nonfibrotic infected mice) (Figure 3A and Supplemental Figure 4). Two out of 27 genes were upregulated and 25 out of 27 genes were downregulated in fibrotic infected mice (Figure 3A). Many downregulated genes fell into the categories of inflammatory responses and cytokines and chemokines (e.g., *Tnf*α, *Il1*, *Cxcl1*), antimicrobial peptides (e.g., *Camp*, *Ctsg*, *Lcn2*), and Toll-like receptors (TLRs) and Nod-like receptors (NLRs) used to sense pathogens (e.g., *Nlrp3*, *Cd14*, *Tlr2*) (Figure 3B). Interestingly, several apoptosis-related genes were also downregulated in fibrotic mice after infection (Figure 3B). The 2 genes that were upregulated fell into the categories of TLR and other pattern recognition receptor (PRR) signaling molecules (*Zbp1*, *Irf7*) (Figure 3B). To validate these findings, we measured via ELISA and reverse transcription quantitative PCR (RT-qPCR) proinflammatory cytokines IL-1β and TNF-α and neutrophil chemokines CXCL1 and CXCL2 in whole lung homogenate and lung cells isolated from infected fibrotic and nonfibrotic mice. IL-1β, TNF-α, CXCL1, and CXCL2 were significantly decreased in fibrotic mice compared with nonfibrotic mice after infection, both at the protein and mRNA levels (Figure 3, C and D). We also measured IL-1β and TNF-α in the spleens of fibrotic mice and found the levels of both cytokines increased compared with levels in nonfibrotic mice, correlating with the elevated splenic bacterial burden (Supplemental Figure 5, A and B). Taken together, these findings point to a broad impairment in immune responses to bacterial lung infections after fibrosis.

### Immune cell landscape and neutrophil recruitment are altered after fibrotic lung disease.

**Figure 4:**
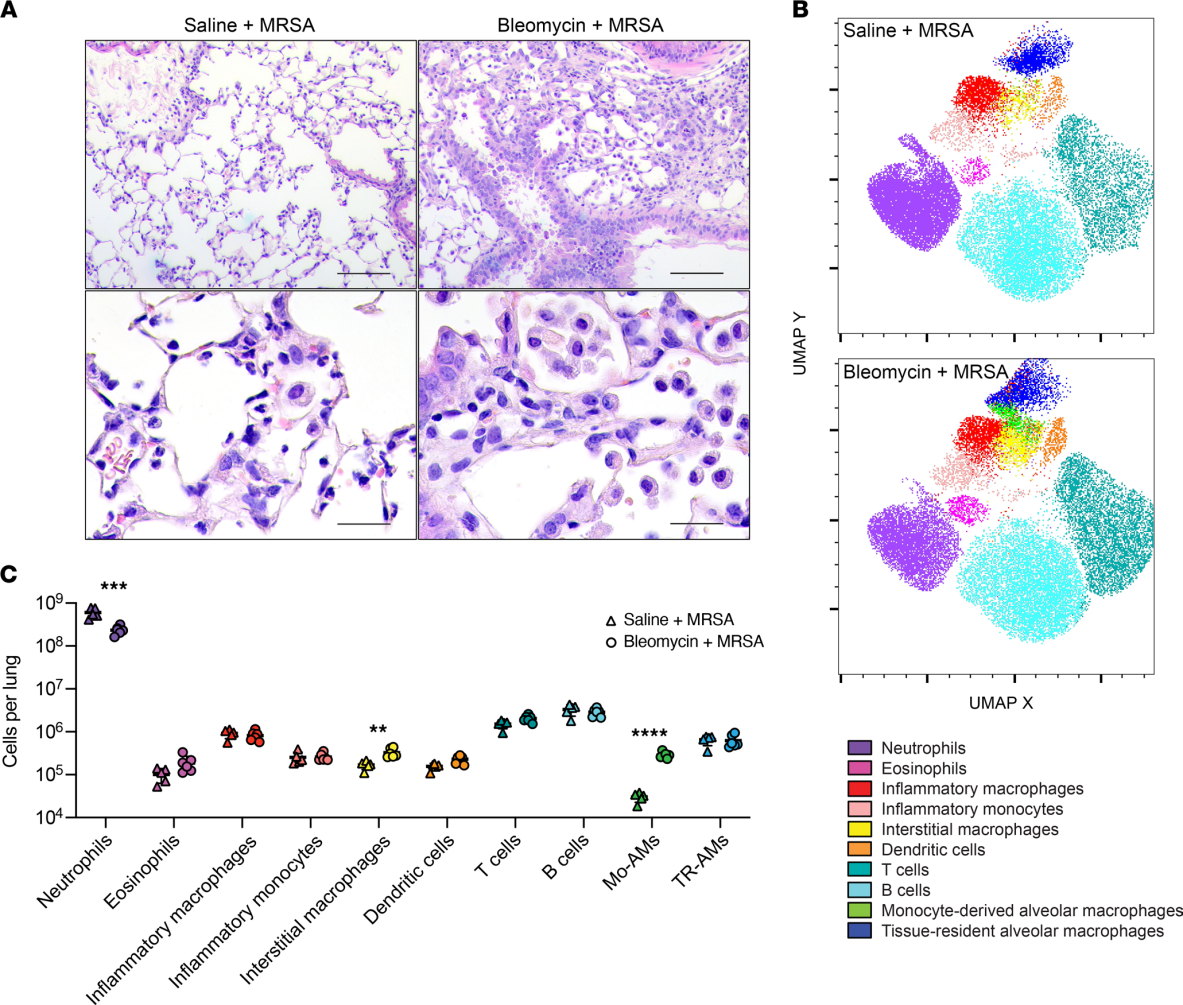
Immune cell landscape and neutrophil recruitment to MRSA are altered 21 days after bleomycin-induced lung fibrosis. (**A**) Lung sections from mice treated with saline + MRSA or bleomycin + MRSA and stained with H&E. Scale bars: 100 μm (top row), 25 μm (bottom row). (**B**) UMAP multidimensional reduction of CD45^+^ lung cells after saline + MRSA or bleomycin + MRSA treatment. (**C**) Quantification of lung immune cell types identified via flow cytometry from mice treated with saline + MRSA (*n* = 5) or bleomycin + MRSA (*n* = 6). Representative of 2 independent experiments. Data represent the means ± SD. Statistical analysis by unpaired Student’s *t* test. **P < 0.01, ***P < 0.001, ****P < 0.0001. Mo-AMs, monocyte-derived alveolar macrophages; TR-AMs, tissue-resident alveolar macrophages.

Based on our observation of decreased CXCL1 and CXCL2 in fibrotic lungs after infection, we hypothesized that immune cell recruitment to the lungs of these mice might be impaired. To assess this, we first used H&E staining to visualize immune cell morphology and localization within the fibrotic and nonfibrotic lungs after infection. In lung sections from nonfibrotic infected mice, we observed an infiltrate of inflammatory cells, many of which morphologically resembled neutrophils with segmented nuclei (Figure 4A). Upon assessment of lung sections from fibrotic infected mice, we also observed increased inflammatory cell infiltrates; however, many of these cells resembled large activated macrophages, and fewer cells with segmented nuclei were seen (Figure 4A). We then quantified immune cell populations present in the lungs of fibrotic and nonfibrotic mice after MRSA infection. We isolated total lung cells from infected fibrotic and nonfibrotic mice via collagenase digestion, and the resulting single-cell suspension was stained for flow cytometry. Uniform manifold approximation and projection (UMAP) was used to dimensionally reduce and visualize the data set (Figure 4B), and numbers of various cell types were quantified (Figure 4C and Supplemental Figures 6 and 7). Like what has been previously reported, we observed a significant increase in the numbers of monocyte-derived alveolar macrophages as well as interstitial macrophages in fibrotic lungs (Figure 4C and Supplemental Figure 6) (19, 20). Interestingly, we also observed a significant difference in the number of neutrophils recruited after infection, which were ~2.6-fold decreased in fibrotic mice compared with nonfibrotic mice (Figure 4C and Supplemental Figure 6). Numerous reports have shown that a robust response to *S*. *aureus* is largely mediated by neutrophils and that loss or depletion of neutrophils dramatically impairs clearance of staphylococcal pneumonia (21–25). The numbers of inflammatory monocytes and macrophages recruited after infection were similar between fibrotic and nonfibrotic mice (Figure 4C and Supplemental Figure 6). In addition, we did not observe a significant difference in monocyte chemokine CCL2 levels in the lungs of infected fibrotic and nonfibrotic mice (Supplemental Figure 8). To determine if other bacterial stimuli might elicit the same neutrophil response as MRSA after fibrosis, we oropharyngeally treated fibrotic and nonfibrotic mice with LPS and measured neutrophil recruitment to the lungs 24 hours after treatment. Interestingly, we did not observe a difference in the numbers of neutrophils present in the lungs of saline + LPS– and bleomycin + LPS–treated mice (Supplemental Figure 9).

### Restoring neutrophil recruitment does not rescue bacterial clearance, probably because of impaired bacterial killing.

**Figure 5:**
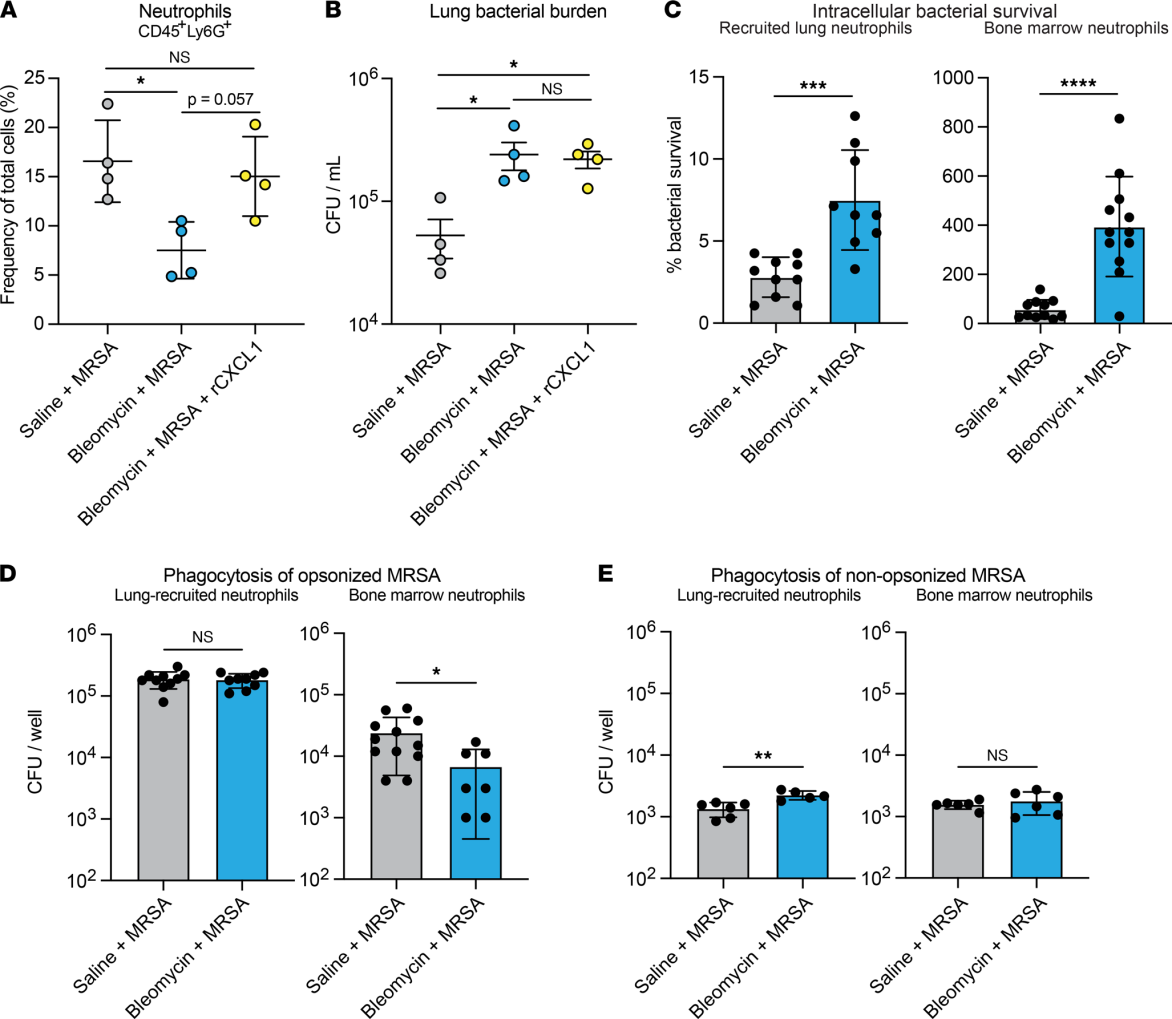
Neutrophils from fibrotic mice have impaired intracellular bacterial killing. (**A**) Quantification of neutrophils isolated from mice treated with saline + MRSA (*n* = 4), bleomycin + MRSA (*n* = 4), or bleomycin + MRSA + rCXCL1 (*n* = 4) (1 × 10^7^ CFU, 21 days after bleomycin). Data from 1 experiment. (**B**) Lung bacterial burden measured from mice in **A**. (**C**) Intracellular bacterial survival after uptake and killing of opsonized MRSA by lung and bone marrow neutrophils infected with MRSA ex vivo. Percentage survival is calculated by dividing the intracellular CFU quantified after 2 hours by the intracellular CFU quantified after 30 minutes and is representative of the bacterial killing by neutrophils. Dots represent technical replicates of pooled cells (lung: saline *n* = 10, bleomycin *n* = 9; bone marrow: saline *n* = 11, bleomycin *n* = 11). Lung cells from 5–6 mice per group; bone marrow cells from 2–3 mice per group. (**D** and **E**) Phagocytosis of opsonized (**D**) or nonopsonized (**E**) MRSA after 30 minutes by lung and bone marrow neutrophils infected ex vivo. Dots represent technical replicates of pooled cells (lung opsonized: saline *n* = 9, bleomycin *n* = 9; bone marrow opsonized: saline *n* = 11, bleomycin *n* = 7; lung nonopsonized: saline *n* = 6, bleomycin *n* = 5; bone marrow nonopsonized: *n* = 6). Lung cells from 5–6 mice per group; bone marrow cells from 2–3 mice per group. For all experiments: Data are representative of 2 or more independent experiments unless otherwise specified. Data represent the means ± SD. Statistical analysis by 1-way ANOVA with Tukey’s multiple comparisons (**A** and **B**) or unpaired Student’s *t* test (**C**–**E**). *P < 0.05, **P < 0.01, ***P < 0.001, ****P < 0.0001.

Because of the well-established role of neutrophils in the clearance of staphylococcal lung infections, we hypothesized that increasing neutrophil recruitment to the lungs of fibrotic mice by treating with recombinant CXCL1 (rCXCL1) during infection would rescue bacterial clearance. We found that treatment of fibrotic mice with rCXCL1 did increase lung neutrophils to the level observed in MRSA-infected nonfibrotic mice (Figure 5A). Surprisingly, this did not correlate with increased bacterial clearance, as mice treated with bleomycin + MRSA + rCXCL1 had similar bacterial burden as mice treated with bleomycin + MRSA, both of which were significantly increased compared with that of mice treated with saline + MRSA (Figure 5B). Together, these data suggest that the absolute number of neutrophils recruited to the lungs of fibrotic mice after infection does not determine bacterial clearance, suggesting an intrinsic neutrophil functional defect. Based on these findings, we next sought to determine whether neutrophils in fibrotic mice exhibit defects in antibacterial functions. We measured MRSA survival after ingestion using neutrophils isolated from lungs and bone marrow of fibrotic and nonfibrotic mice and determined that intracellular bacterial survival was significantly increased in neutrophils isolated from both the bone marrow and the lungs of fibrotic mice compared with nonfibrotic mice, indicating that neutrophil intracellular killing of MRSA is impaired after fibrosis (Figure 5C and Supplemental Figure 10). We next measured phagocytosis of both opsonized and nonopsonized MRSA by lung and bone marrow neutrophils. While we observed impaired phagocytosis of opsonized MRSA by bone marrow neutrophils from fibrotic mice, this defect was not evident in lung-recruited neutrophils (Figure 5D and Supplemental Figure 10). Additionally, there was no difference in the phagocytosis of nonopsonized MRSA by bone marrow neutrophils and a small, but significant, increase by lung-recruited neutrophils from fibrotic mice compared with nonfibrotic mice (Figure 5E). Therefore, we hypothesized that the defect in neutrophil antibacterial responses in fibrotic mice was largely due to impaired intracellular killing.

### Neutrophil antimicrobial effector functions are impaired after fibrotic lung disease.

**Figure 6:**
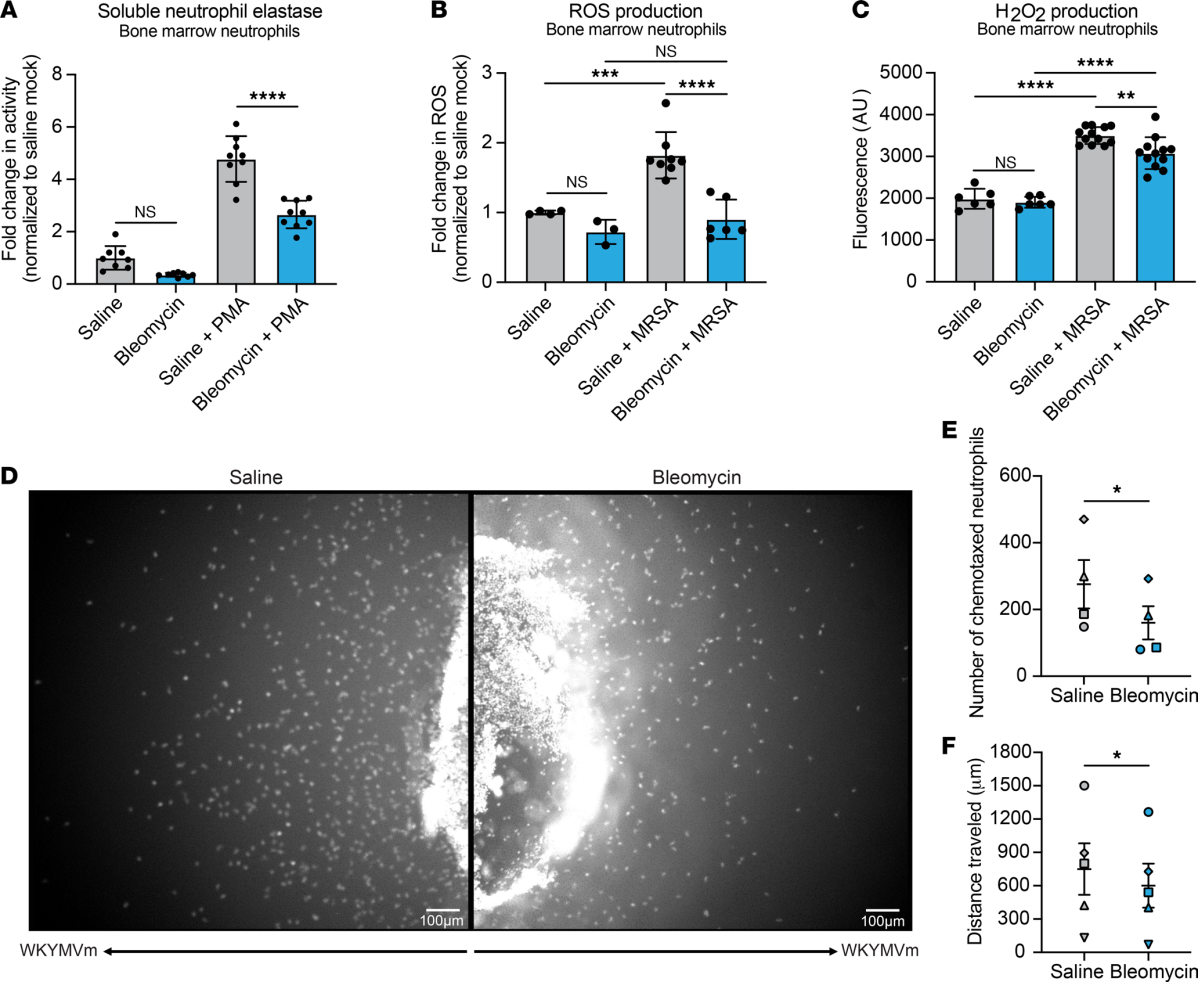
Fibrosis inhibits NE release, ROS and H_2_O_2_ production, and neutrophil chemotaxis. (**A**) NE release by bone marrow neutrophils (*n* = 8). Cells from 2–3 mice per group. Data are representative of 2 independent experiments. (**B**) ROS production by bone marrow neutrophils (saline *n* = 4, bleomycin *n* = 3, saline + MRSA *n* = 8, bleomycin + MRSA *n* = 6). Cells from 4–6 mice per group. Data from 2 combined experiments. (**C**) H_2_O_2_ production by bone marrow neutrophils (uninfected *n* = 6, saline + MRSA *n* = 12, bleomycin + MRSA *n* = 11). Cells from 2–3 mice per group. Data are representative of 2 independent experiments. (**A**–**C**) Dots represent technical replicates of pooled cells. Statistical analysis by 1-way ANOVA with Tukey’s multiple comparisons. Data represent the means ± SD. **P < 0.01, ***P < 0.001, ****P < 0.0001. (**D**) Under agarose chemotaxis assay measuring bone marrow neutrophil migration toward formyl peptide receptor (FPR) agonist WKYMVm (100 nM) for 3 hours with 20-second time intervals. Representative endpoint images of the migrating neutrophils from saline or bleomycin-treated mice. Five out of 6 experiments performed showed inhibited migration in neutrophils from fibrotic mice compared with neutrophils from nonfibrotic mice, while 1 out of 6 experiments showed the opposite effect. (**E** and **F**) Bone marrow neutrophils from saline- and bleomycin-treated mice were allowed to migrate under agarose toward WKYMVm (100 nM) for 1.5 to 4 hours. Plots showing the number of neutrophils that left the well (**E**) and distance traveled by the chemotaxing neutrophils (**F**) are presented. Data represent mean ± SEM from 4–5 independent experiments. Data points with identical shape represent data collected from the same experiment with matched migration time. *P < 0.05 when compared with saline group (paired 2-tailed Student’s *t* test).

Because we observed similar trends in bacterial killing by lung and bone marrow neutrophils, we performed further ex vivo mechanistic experiments with neutrophils isolated from bone marrow. To verify that the impaired intracellular killing was not a function of decreased neutrophil survival after infection, we measured neutrophil cell death via lactate dehydrogenase (LDH) assay and observed no significant difference in survival of neutrophils isolated from fibrotic and nonfibrotic mice after infection (Supplemental Figure 11A). We next evaluated mechanisms of intracellular killing by measuring neutrophil elastase (NE) release, reactive oxygen species (ROS) production, and hydrogen peroxide production. Interestingly, NE, ROS, and hydrogen peroxide were all decreased in neutrophils from fibrotic mice, suggesting a broad impairment of intracellular killing mechanisms (Figure 6, A–C). We also measured the production of nitric oxide by neutrophils but observed no difference between neutrophils isolated from fibrotic mice and neutrophils isolated from nonfibrotic mice (Supplemental Figure 11B). We hypothesized that the decreased production of ROS and hydrogen peroxide was due to the downregulation of enzymes involved in generation of the respiratory burst and free radicals, so we measured the expression of genes encoding nitric oxide synthase 2, NADPH oxidase 2, myeloperoxidase, and superoxide dismutase 1 and 2 (*Nos2*, *Nox2*, *Mpo*, *Sod1*, and *Sod2*, respectively). We observed no significant difference in the expression of any of these enzymes, indicating that ROS and hydrogen peroxide production in fibrotic mice is not inhibited at the transcriptional level for these genes (Supplemental Figure 11C). We hypothesized that the decreased release of NE by neutrophils from fibrotic mice is due to a downregulation of antimicrobial peptide expression and measured transcription of genes encoding cathelicidin, lipocalin-2, cathepsin G, and NE (*Camp*, *Lcn2*, *Ctsg*, and *Elane*, respectively). There was no difference in *Camp*, *Lcn2*, and *Ctsg*, but interestingly, expression of *Elane* was upregulated in neutrophils from fibrotic mice (Supplemental Figure 11D). We also used under agarose chemotaxis assays to measure the migration of neutrophils in response to WKYMVm, a formyl peptide receptor (FPR) agonist. FPR recognizes formylated peptides released by bacteria and allows neutrophils to migrate directionally, or chemotax, in response to infection by *S*. *aureus* and other bacterial species (26, 27). We observed decreased chemotaxis toward WKYMVm by neutrophils isolated from fibrotic mice compared with nonfibrotic mice (Figure 6D). Fewer neutrophils from fibrotic mice migrated toward the WKYMVm gradient compared with neutrophils from nonfibrotic mice, with minimal migration from the back side of the wells in both groups, indicating a chemotactic response (Figure 6, D and E, and Supplemental Videos 1–4). In addition, the distance traveled by individual neutrophils from fibrotic mice was decreased compared with that of neutrophils from nonfibrotic mice (Figure 6F, Supplemental Videos 5 and 6, and Supplemental Table 1). Taken together, these data highlight several mechanisms by which neutrophil antimicrobial functions are impaired after the development of fibrosis.

### Neutrophil functions are differentially regulated by soluble mediators from fibrotic lungs.

**Figure 7:**
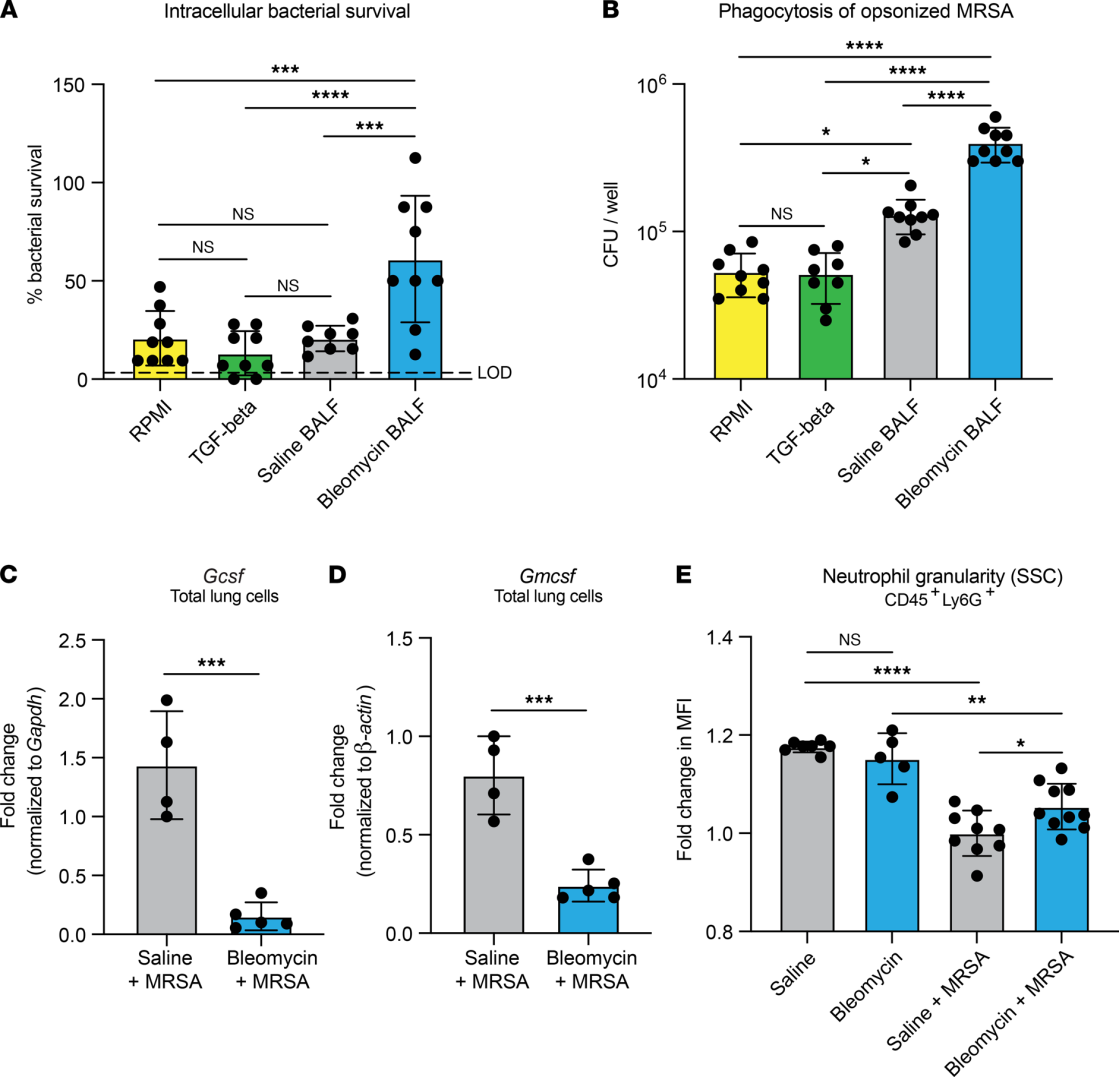
Soluble mediators in fibrotic lungs likely play a role in impaired effector function. (**A**) Intracellular bacterial survival after uptake and killing of opsonized MRSA by bone marrow neutrophils from naive mice treated for 4 hours with complete RPMI, complete RPMI + TGF-β (2 ng/mL), or BALF from saline- or bleomycin-treated mice collected in complete RPMI. BALF was collected from mice 21 days after saline or bleomycin. Percentage survival is calculated by dividing the intracellular CFU quantified after 2 hours by the intracellular CFU quantified after 30 minutes and is representative of the bacterial killing by neutrophils. (**B**) Phagocytosis of opsonized MRSA by cells described in **A**. (**A** and **B**) Representative of 2 independent experiments; dots represent technical replicates of pooled cells (*n* = 9). Cells from 2 mice per group. Data represent the means ± SD. Statistical analysis by 1-way ANOVA with Tukey’s multiple comparisons. *P < 0.05, ***P < 0.001, ****P < 0.0001. (**C** and **D**) *Gcsf* (**C**) and *Gmcsf* (**D**) measured via RT-qPCR of total lung cells isolated from mice treated with saline + MRSA (*n* = 4) or bleomycin + MRSA (*n* = 5). Representative of 2 independent experiments. Data represent the means ± SD. Statistical analysis by unpaired Student’s *t* test. ***P < 0.001. (**E**) Neutrophil granularity as measured by mean fluorescence intensity of cellular side-scatter (SSC). Neutrophils were analyzed 21 days after treatment with saline (*n* = 7), bleomycin (*n* = 5), saline + MRSA (*n* = 9), or bleomycin + MRSA (*n* = 10). Mice were infected with MRSA 24 hours before analysis. Data from 2 combined experiments. Data represent the means ± SD. Statistical analysis by 1-way ANOVA with Tukey’s multiple comparisons. *P < 0.05, **P < 0.01, ****P < 0.0001.

We next sought to determine the effect of extracellular mediators in fibrotic lungs on neutrophil effector functions and hypothesized that bronchoalveolar lavage fluid (BALF) from fibrotic mice would have an inhibitory effect on neutrophil antimicrobial activity. To test this, we treated neutrophils isolated from bone marrow of naive mice with BALF collected from uninfected mice treated with saline or bleomycin (saline BALF or bleomycin BALF, respectively). We also treated a group of neutrophils with TGF-β, as many effects of fibrotic lung disease and pathology are attributed to TGF-β production and signaling. Following ex vivo stimulation with BALF and TGF-β, intracellular bacterial survival was measured. Treatment with RPMI control media, TGF-β, and saline BALF induced similar killing responses (20.8%, 13.2%, and 20.6% bacterial survival, respectively). However, treatment with bleomycin BALF significantly increased bacterial survival (61.1%), indicating that MRSA killing by neutrophils is inhibited by soluble mediators in the fibrotic lung environment (Figure 7A and Supplemental Figure 12). Stimulation with saline BALF increased phagocytosis of opsonized MRSA compared with treatment with RPMI control media and TGF-β (Figure 7B and Supplemental Figure 12). Surprisingly, treatment of neutrophils with bleomycin BALF resulted in the highest levels of phagocytosis, significantly increased over even the saline BALF–treated neutrophils (Figure 7B and Supplemental Figure 12). We next postulated that, in addition to inhibitory factors present at baseline in the fibrotic lung, production of inflammatory mediators responsible for maturation and function of neutrophils may be impaired in fibrotic mice during infection. We measured the expression of *Gcsf* and *Gmcsf*, 2 growth factors well established in their role of promoting maturation and function of neutrophils at sites of inflammation (28–31), and found the expression of both mediators decreased in total lung cells isolated from fibrotic mice compared with nonfibrotic mice (Figure 7, C and D). In addition, we measured the granularity of neutrophils recruited to the lungs of fibrotic and nonfibrotic mice after infection by flow cytometric evaluation of cellular side-scatter. Previous studies show that decreased granularity corresponds with increased age and activation of neutrophils (32–35). We found that neutrophils recruited to nonfibrotic lungs after infection were significantly less granular than neutrophils recruited to fibrotic lungs, suggesting that functional maturation of neutrophils in fibrotic mice is impaired (Figure 7E).

### Macrophage phagocytosis but not killing of MRSA is impaired after fibrotic lung disease.

**Figure 8:**
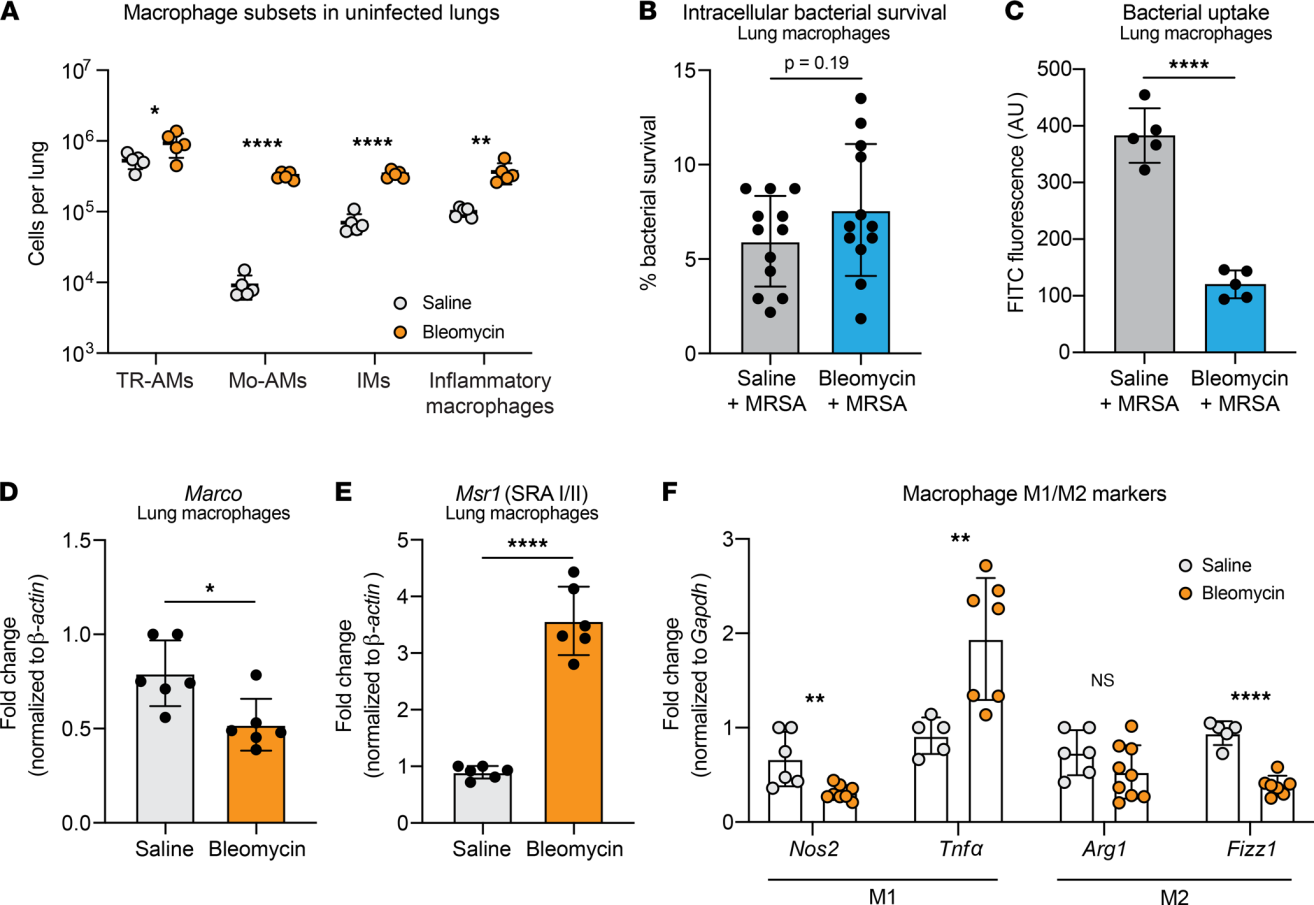
Fibrosis inhibits lung macrophage phagocytosis. (**A**) Lung macrophage populations after bleomycin or saline treatment as quantified via flow cytometry. *n* = 5–6 mice per group. *P < 0.05, **P < 0.01, ****P < 0.0001. (**B**) Intracellular bacterial survival after uptake and killing of opsonized MRSA by lung macrophages infected with MRSA ex vivo. Percentage survival is calculated by dividing the intracellular CFU quantified after 2 hours by the intracellular CFU quantified after 30 minutes and is representative of the bacterial killing by macrophages. Dots represent technical replicates of pooled cells (*n* = 12). Cells from 2–3 mice per group. (**C**) Ex vivo phagocytosis of heat-killed FITC-conjugated MRSA by total lung macrophages isolated from bleomycin- or saline-treated mice (2–3 mice per group). Dots represent technical replicates of pooled cells (*n* = 5). ****P < 0.0001. (**D** and **E**) *Marco* (**D**) and *Msr1* (**E**) expression by uninfected lung macrophages isolated from mice 21 days after saline or bleomycin treatment. Dots represent technical replicates of pooled cells (*n* = 6). Cells from 2–3 mice per group. Data from 2 combined experiments. *P < 0.05, ****P < 0.0001. (**F**) Expression of macrophage polarization markers *Nos2*, *Tnfα*, *Arg1*, and *Fizz1* by uninfected lung macrophages isolated from mice 21 days after saline or bleomycin treatment. Dots represent technical replicates of pooled cells (*n* = 5–9). Cells from 2–3 mice per group. Data from 2 combined experiments. **P < 0.01, ****P < 0.0001. For all figures: Representative of 2 independent experiments unless otherwise specified. Data represent the means ± SD. Statistical analysis by unpaired Student’s *t* test.

While we observed no difference in the recruitment of inflammatory monocytes and macrophages following infection of fibrotic mice, we hypothesized that the antimicrobial functions of lung-resident macrophages may be altered after fibrosis. In accordance with previous studies (19, 20), we found that fibrosis alters the macrophage populations in the lung, with significant increases in the numbers of TR-AMs, Mo-AMs, interstitial macrophages (IMs), and inflammatory macrophages 21 days after bleomycin treatment alone (Figure 8A). We next evaluated the antibacterial functions of the total macrophage population by measuring intracellular bacterial survival and phagocytosis of MRSA. We found that, while bacterial survival and therefore intracellular killing of MRSA were unchanged (Figure 8B and Supplemental Figure 13A), phagocytosis of MRSA was significantly impaired in macrophages from fibrotic mice (Figure 8C and Supplemental Figure 13B). Interestingly, this was specific to the uptake of nonopsonized MRSA, as phagocytosis of opsonized MRSA was not different (Supplemental Figure 13, A and C). We hypothesized that changes in the expression of macrophage scavenger receptors MARCO and SR-A might mediate the impaired phagocytosis in macrophages from fibrotic mice, as both scavenger receptors are important for the uptake of nonopsonized *S*. *aureus* (36–38). Indeed, we measured decreased expression of the gene encoding MARCO (*Marco*) by macrophages from fibrotic mice (Figure 8D). Conversely, we observed increased expression of the gene encoding SR-A (*Msr1*) by macrophages from fibrotic mice compared with nonfibrotic mice (Figure 8E). As expected, there was no change in the expression of receptors driving uptake of opsonized MRSA (*Cr3* and *Fcγr1*) (Supplemental Figure 13, D and E). Macrophage polarization can affect phagocytosis, so we evaluated the expression of a series of M1/M2 macrophage markers (39) to determine if macrophages from fibrotic mice have a less inflammatory, more reparative M2-like phenotype. While we observed decreased expression of M1 marker *Nos2* in macrophages from fibrotic mice, M1 marker *Tnfα* was upregulated in macrophages from fibrotic mice compared with nonfibrotic mice (Figure 8F). We measured no change in M2 marker *Arg1* but decreased expression of M2 marker *Fizz1* in macrophages from fibrotic mice (Figure 8F).

## Discussion

Respiratory infection is known to cause increased mortality in patients with IPF, but the mechanism(s) responsible for this phenomenon are largely unknown (9, 11). Few experimental studies exist that mechanistically tie infection to progression of fibrotic lung disease or fibrotic lung injury to altered susceptibility to infection with clinically relevant bacterial pathogens (40–43). In the present study, we highlight a potentially novel observation in pulmonary fibrosis, showing that fibrosis impairs antibacterial immune responses in neutrophils and macrophages, resulting in increased mortality after MRSA infection.

Our initial observations determined that fibrosis induces a broad downregulation in the inflammatory response to MRSA, characterized by a reduction in cytokine and chemokine production. Because we measured decreased levels of neutrophil chemokines CXCL1 and CXCL2, we hypothesized that immune cell recruitment to the lung in response to infection may be altered after fibrotic lung injury. In accordance with these findings, we found fewer neutrophils are recruited to the lungs of fibrotic mice after infection compared with nonfibrotic mice. The numbers of other recruited immune cells such as inflammatory macrophages and monocytes were unchanged.

As neutrophils have been shown to play a critical role in the clearance of staphylococcal pneumonia (21–25), we hypothesized that boosting neutrophil recruitment by treating with rCXCL1 to supplement the defect in chemokine production after fibrosis would result in greater bacterial clearance. Interestingly, we did not see a reduction in bacterial burden after rCXCL1 treatment, suggesting that the absolute number of neutrophils recruited to the lung after infection of fibrotic mice does not correlate with bacterial clearance. Therefore, we hypothesized that neutrophils exhibit a functional defect in antibacterial responses after fibrotic lung injury. Indeed, we measured impaired intracellular killing likely mediated by inhibition of NE release and oxidative radical production, suggesting that the bacterial clearance defect observed after fibrotic lung injury could be due to functional impairments in neutrophil killing capacity. We have ruled out transcriptional effects on respiratory burst enzymes and antimicrobial peptide production to explain the defective intracellular killing in neutrophils from fibrotic mice, and future studies will focus on elucidating the mechanisms of these posttranscriptional regulations. We also observed decreased chemotaxis toward the FPR agonist WKYMVm by neutrophils from fibrotic mice. While we were able to restore the total number of neutrophils recruited to the lung of fibrotic mice with rCXCL1 treatment, chemotaxis within the local environment of the lung is critical for neutrophil localization at the site of infection. Therefore, decreased migration toward WKYMVm is indicative of another impairment in neutrophil antimicrobial responses. To our knowledge, this is the first study to outline intrinsic functional alterations to neutrophil immune responses after fibrotic lung disease. Of added interest is our observation that neutrophils isolated from fibrotic mice express higher levels of the serine protease NE, encoded by the gene *Elane*, compared with neutrophils isolated from nonfibrotic mice. While elevated *Elane* did not affect MRSA intracellular killing or NE release by bone marrow neutrophils following ex vivo stimulation, NE has been linked to fibrotic development; NE-deficient mice are resistant to bleomycin-induced fibrosis and show decreased production of pathogenic TGF-β relative to wild-type mice (44). Additionally, NE is known to promote fibroblast differentiation into myofibroblasts (45). Thus, these studies suggest that altered neutrophil function in fibrotic mice may contribute to lung pathology at baseline as well as impaired host defense after infection.

While we demonstrated that lung and bone marrow neutrophils from fibrotic mice have intrinsic defects in intracellular killing of MRSA, we hypothesized that soluble mediators within the fibrotic lung microenvironment may also play a role in modulating neutrophil function. Indeed, we found that treatment of naive bone marrow neutrophils with BALF from fibrotic mice inhibited intracellular killing of MRSA compared with treatment with saline BALF (20.6% versus 61.1% bacterial survival after 2 hours, respectively). Interestingly, killing by bone marrow neutrophils from fibrotic mice (as described in Figure 5C) was even more impaired, as MRSA survival was 394.7%, indicating that the bacteria were able to replicate intracellularly. This suggests that inhibition of intracellular killing by soluble mediators in the fibrotic lung is likely additive to cell-intrinsic effects mediating the downregulation of antimicrobial responses. Paradoxically, we found that treatment of naive neutrophils with bleomycin BALF increased phagocytosis of nonopsonized MRSA compared with treatment with saline BALF. In contrast to our intracellular killing findings, we demonstrated in Figure 5D that neutrophils isolated from the bone marrow of fibrotic mice had a defect in phagocytosis of opsonized MRSA while neutrophils isolated from fibrotic lungs did not. This could suggest that soluble mediators in the fibrotic lung environment that promote neutrophil phagocytosis can rescue the phagocytic defect (but not the killing defect) observed in bone marrow–derived neutrophils from fibrotic mice once the neutrophils enter the lung.

Once neutrophils are recruited to the site of infection, inflammatory mediators in the local environment, such as TNF-α, G-CSF, and GM-CSF, are necessary to promote the activation of neutrophils in inflamed tissues. We observed decreased lung expression of *Tnfα*, *Gcsf*, and *Gmcsf*, as well as increased granularity of neutrophils recruited to fibrotic lungs after infection, suggesting that functional maturation of neutrophils in fibrosis is impaired. These data, in addition to the impaired intracellular killing of MRSA following treatment of naive neutrophils with bleomycin BALF, point to a role for soluble mediators within the lung microenvironment dictating neutrophil antibacterial functions in addition to cell-intrinsic defects.

Another unexpected observation was that ex vivo antibacterial responses are impaired in neutrophils isolated from both the lung and the bone marrow of fibrotic mice, indicating that fibrosis affects neutrophil function outside of the lung microenvironment. Because of this, it is interesting to speculate about the effects of fibrosis on the development, aging, and clearance of neutrophils within both the bone marrow niche and inflamed tissue sites. Neutrophil release from the bone marrow into circulation is a complex process regulated in part by a balance of chemokine signaling (46). Neutrophils spend several hours circulating throughout the body, during which the cells age and eventually return to the bone marrow for clearance. Adequate clearance of neutrophils from circulation is essential for the appropriate function of hematopoietic cells within the bone marrow niche (32). During acute inflammation, however, aged neutrophils do not return to the bone marrow for clearance and instead migrate toward the injured tissue and exhibit more potent inflammatory responses compared with those of non-aged neutrophils, suggesting that aging is an important step in the development of neutrophil immune functions (47, 48). In IPF, it has been shown that increased neutrophils in peripheral blood are predictive of poor outcomes, including acute exacerbation, disease progression, and death (49–51). It is possible that the mechanism by which fibrosis alters bone marrow neutrophil antibacterial functions is related to what is driving increased neutrophil levels in the circulation of IPF patients, possibly altering egress from the bone marrow, aging in circulation, or eventual clearance. Therefore, future studies should seek to fully understand the effects of fibrosis on neutrophil function and mobilization between the bone marrow and peripheral blood.

In addition to our findings showing that neutrophil killing was impaired in fibrosis, we also observed defects in macrophage phagocytosis of MRSA in fibrotic mice. This is likely due, at least in part, to the downregulation of the macrophage scavenger receptor MARCO, which has been shown to be important in the uptake of nonopsonized *S*. *aureus* (36–38). Interestingly, however, macrophage scavenger receptor SR-A was significantly upregulated in lung macrophages from fibrotic mice, suggesting either that the function of MARCO is more important than SR-A in our model or that a mechanism unrelated to scavenger receptor expression mediates impaired phagocytosis by macrophages in fibrotic mice. To this end, we assessed the polarization of lung macrophages in fibrosis to determine whether they were skewed toward an antiinflammatory M2-like phenotype. Surprisingly, macrophages in fibrotic lungs were not clearly skewed toward an M2-like phenotype, indicating that macrophage polarization is not driving the phagocytic impairment. Like other researchers (19, 20), we observed significant differences in the composition of the total macrophage population of the fibrotic lung, with increases in TR-AMs, Mo-AMs, IMs, and inflammatory macrophages at baseline (Figure 8A). It is possible that the defect in MRSA phagocytosis can be localized to one specific macrophage subset and that our experiments evaluating the total lung macrophage population are not granular enough to identify a mechanism underlying the phagocytic impairment. Future studies will focus on evaluating the function of individual lung macrophage populations in fibrosis.

To summarize the present findings, we have identified a potentially novel mechanism by which infection results in poor outcomes after fibrosis. This mechanism is driven by defects in the immune response to MRSA conferred by fibrotic lung injury, resulting in decreased clearance of bacteria from the lung and increased morbidity and mortality. These defects include impaired production of proinflammatory cytokines and chemokines and a concurrent decrease in neutrophil recruitment to the lung following infection. This recruitment defect may be specific to MRSA or other Gram-positive pathogens, as neutrophil recruitment following LPS treatment is not decreased in fibrotic mice. Interestingly, this aligns with clinical data showing that Gram-positive pathogens such as *S*. *aureus* and *Streptococcus* *pneumoniae* are frequently isolated from IPF patients and that increases in the lung burden of *Staphylococcus* and *Streptococcus* species are associated with progressive IPF (8–11, 13, 16). While neutrophil recruitment could be rescued with the treatment of rCXCL1 at the time of MRSA infection, this did not result in decreased lung bacterial burdens. We have demonstrated that neutrophils from fibrotic mice exhibit a distinct impairment in intracellular bacterial killing likely driven by impaired NE release and oxidative radical production. In addition, we also observed a defect in lung macrophage phagocytosis in fibrotic mice. The present study serves as a likely model delineating why staphylococcal infections drive increased morbidity and mortality in IPF. Together our study highlights the need to fully understand the impact of fibrosis on innate immune cell function, as this may be a major factor mediating survival of patients with IPF experiencing pulmonary infection.

## Methods

### Mice and in vivo inoculations.

C57BL/6J mice, 6 to 8 weeks old, from The Jackson Laboratory were used for all experiments. Mice were housed in specific pathogen–free conditions at the University of Michigan Animal Care Facility for the duration of experiments. All animal experiments were performed with approval by the University of Michigan IACUC.

Bleomycin, MRSA, rCXCL1, and LPS were administered to mice via oropharyngeal inoculation. Mice were anesthetized with a mixture of ketamine and xylazine and 50 μL of bleomycin, MRSA, and/or rCXCL1, and LPS was pipetted into the back of the throat while the tongue was pulled forward and inoculum was aspirated into the lungs.

Experiments were terminated 21 days after bleomycin or saline treatment (unless otherwise specified in figure legends), and mice were euthanized by inhalation of CO_2_.

### Bleomycin, MRSA, recombinant CXCL1, and LPS.

Pharmaceutical grade bleomycin was obtained from the University of Michigan Hospital and diluted in sterile PBS. Bleomycin was administered at a concentration of approximately 0.016 U per 20-gram mouse.

MRSA strain USA300 was obtained from the Network of Antimicrobial Resistance in *Staphylococcus aureus*. To prepare bacterial cultures for infections, MRSA was grown overnight at 37°C with constant shaking in Nutrient Broth (BD Difco). CFU were determined by optical density relative to a standard curve for an inoculation concentration of approximately 1 × 10^7^ or 7 × 10^7^ CFU per mouse. Experiments were terminated 24 hours postinfection unless otherwise specified in figure legends.

For experiments where opsonized MRSA was used, bacteria were resuspended to a concentration of 1 × 10^8^ CFU/mL and treated with 1 μL of 4 mg/mL anti–*S*. *aureus* polyclonal antibody (catalog ab20920) (Abcam) per 3 mL of bacterial culture. Cultures were rocked at 37°C for 30 minutes and then used for subsequent ex vivo infections.

Recombinant CXCL1 (R&D Systems, Bio-Techne) was administered to mice at a concentration of 1 μg/mouse.

LPS from *Pseudomonas aeruginosa* (MilliporeSigma) was administered to mice at a concentration of 25 μg/mouse as previously described (52).

### CFU, cytokine analysis, and hydroxyproline assay.

After euthanasia, lungs were perfused with 3 mL sterile PBS. Lung and spleen tissue were homogenized in 1 mL sterile PBS with cOmplete protease inhibitor (Roche, MilliporeSigma). IL-1β, TNF-α, CXCL1, CXCL2, and CCL2 ELISAs were performed using Duoset ELISA kits (R&D Systems, Bio-Techne) according to manufacturer protocols. Bacterial burden was quantified from lungs and spleen by serially diluting homogenized tissue and plating on Nutrient Agar (BD Difco) for 24 hours at 37°C. Hydroxyproline assays were performed as previously described (53).

CFU, cytokines, and hydroxyproline were quantified 24 hours postinfection unless otherwise specified in figure legends.

### Bronchoalveolar lavage.

Following euthanasia of each mouse, a catheter was inserted into the trachea, and the lungs were flushed with 1 mL of complete RPMI (RPMI + 10% FBS + 1% l-glutamine + 1% penicillin/streptomycin) to collect cytokines, then diluted 1:1 with complete RPMI for neutrophil stimulation.

### Total lung cell preparation, cell marker staining for flow cytometry, and UMAP analysis.

Total lung cells were collected 24 hours postinfection as previously described (53). Briefly, lungs were finely minced with scissors and incubated in a solution containing DMEM, 10% fetal calf serum, 1 mg/mL collagenase (MilliporeSigma), and 30 μg/mL DNase for 35 minutes at 37°C. Following mechanical dispersion, the cell suspension was filtered through a 100 μM filter and centrifuged at 2000*g* for 20 minutes at 4°C with deceleration set to 0 through a 20% Percoll (MilliporeSigma) gradient. Total lung cell counts and viability were determined by trypan blue (Corning) exclusion on a hemocytometer.

After total lung cells were isolated, nonspecific Fc binding was blocked with anti-CD16/32 (catalog 553142) (BD Biosciences) while incubating on ice for 15 minutes, after which primary antibodies were added to cells and incubated on ice for 30 minutes in the dark. The following antibodies were used: Ly6G-FITC (catalog 561105), SiglecF-APC/Cy7 (catalog 565527), MHC II-BV421 (catalog 562564), CD45-BV510 (catalog 563891) (BD Biosciences), Ly6C-PE/Cy7 (catalog 128018), CD24-PerCP/Cy5.5 (catalog 101824), CD64-BV605 (catalog 139323), CD11b-BV650 (catalog 101259), and CD11c-PE/dazzle (catalog 117348) (BioLegend). After staining, cells were washed and fixed in 4% paraformaldehyde.

The UMAP FlowJo plugin was used to dimensionally reduce and visualize data sets. Samples were grouped according to treatment, and a standard number of events from each group were concatenated into separate FCS files, which were then concatenated into a single FCS file to preserve group identity. The UMAP algorithm was run on single FCS files to separate groups based on surface markers. The standardized event numbers were based on pregated CD45^+^ cells. Gating strategies were based on those described by Yu et al. (54).

### Isolation of RNA for RT-qPCR.

Total RNA was isolated from cells using TRIzol (Invitrogen, Thermo Fisher Scientific) according to the manufacturer’s instructions, and RNA concentration was normalized between samples. RT-qPCR was performed using an ABI StepOnePlus real-time thermocycler (Applied Biosystems, Thermo Fisher Scientific) and TaqMan RNA-to-Ct 1-Step Kit (Applied Biosystems, Thermo Fisher Scientific). All primers and probes used in this study are listed in Supplemental Table 2 and were obtained from MilliporeSigma. The QIAGEN RT2 Profiler PCR Array for mouse antibacterial responses was used to evaluate expression of inflammatory genes by total lung cells. RNA from 4 mice per group was pooled and converted to cDNA using the RT2 First Strand Kit (QIAGEN). After cDNA conversion, RT2 SYBR Green qPCR Mastermix (QIAGEN) was added to samples, and samples were loaded onto Profiler Array plates and run according to manufacturer instructions.

### Bone marrow and lung neutrophil isolation and ex vivo functional assays.

Bone marrow neutrophils were isolated from femurs and tibias of bleomycin or saline-treated mice 21 days posttreatment as previously described (52). Briefly, the ends of the bones were removed with scissors, and the marrow was flushed out with DMEM with 10% fetal calf serum using a 23.5 gauge needle. After mechanical dispersion, filtration, and red blood cell lysis, bone marrow was centrifuged at 930*g* for 30 minutes at 25°C with deceleration set to 0 through a Histopaque 1119/1077 (MilliporeSigma) gradient to isolate neutrophils.

Lung neutrophils were isolated 24 hours after recruitment to the airspaces with 1 μg rCXCL1 as described above. Neutrophils were collected via collagenase digestion and isolated using a Histopaque 1119/1077 gradient.

To measure intracellular bacterial survival as a representation of neutrophil intracellular killing, lung and bone marrow neutrophils were infected with MRSA (MOI = 50) that had been opsonized, as described above. Neutrophil samples were plated in duplicate and incubated with opsonized MRSA for 30 minutes to allowed for bacterial uptake, after which extracellular bacteria were killed with lysostaphin (MilliporeSigma). One set of infected cells were lysed with 0.5% saponin to release intracellular bacteria, which were diluted and plated on Nutrient Agar to quantify phagocytosis after 30 minutes. The second set of infected neutrophils were then incubated for 1.5 hours (for a total of 2 hours) to allow time for intracellular killing of the bacteria, after which any remaining extracellular bacteria were again killed with lysostaphin. These neutrophils were lysed with 0.5% saponin, and the intracellular bacteria were diluted and plated as described above to quantify the surviving intracellular bacteria. To calculate percentage bacterial survival, the intracellular bacterial CFU quantified after 2 hours’ total incubation were divided by the intracellular CFU quantified after 30 minutes.

To measure ex vivo phagocytosis, lung and bone marrow neutrophils were infected with opsonized or nonopsonized MRSA (MOI = 50) for 30 minutes, after which extracellular bacteria were killed with lysostaphin. The infected neutrophils were lysed with 0.5% saponin and the intracellular bacteria were diluted and plated and described above. To account for possible differences in the numbers of neutrophils plated between samples, LDH assays were used to account for cell density. The quantified bacterial CFU was then normalized to the LDH value.

For ex vivo NE release assays, an NE activity assay kit (Cayman Chemical) was used. Per well, 400,000 neutrophils were treated with 200 nM PMA for 2 hours, after which supernatant was collected and incubated with elastase-specific substrate (Z-Ala-Ala-Ala-Ala)2Rh110 for 1.5 hours at 37°C. Upon cleavage by NE, (Z-Ala-Ala-Ala-Ala)2Rh110 yields fluorescence detectable at excitation/emission (ex/em) 485 nm/525 nm.

To measure ex vivo ROS production, 200,000 neutrophils were plated in 96-well plates. Neutrophils were infected with MRSA (MOI = 50) for 1.5 hours, after which 100 μL of 200 μM dihydroethidium (Invitrogen, Thermo Fisher Scientific) in plain RPMI was added to each well to a final concentration of 100 μM. Cells were then incubated for 30 minutes at 37°C, and ROS production was measured by reading the fluorescence at ex/em 518 nm/606 nm.

To measure ex vivo hydrogen peroxide production, we plated 200,000 neutrophils in 96-well plates. Neutrophils were infected with MRSA (MOI = 50) for 1.5 hours, and Amplex Red Assays (Invitrogen, Thermo Fisher Scientific) were performed. Briefly, Amplex Red solution (100 μL of 50 μM of Amplex Red reagent and 0.1 U/mL HRP into PBS) was added to each well and incubated for 30 minutes at 37°C. Hydrogen peroxide was measured by taking the fluorescence at ex/em 530 nm/590 nm.

To measure ex vivo survival, LDH assays (Cytotoxicity Detection Kit, Roche, MilliporeSigma) were performed. Briefly, neutrophils were infected with MRSA (MOI = 50) for 2 hours, after which supernatant and dead cells were aspirated, and remaining adherent cells were lysed in a 2% Triton X-100 solution. Cell lysates were used to quantify surviving cells that remained adhered to the plate following the 2-hour infection.

For ex vivo nitric oxide quantification, Griess assays were performed on supernatant from neutrophils infected with MRSA (MOI = 50) for 2 hours. Griess assays measure nitrite levels resulting from the spontaneous oxidation of nitric oxide under physiological conditions.

Under agarose migration assays were performed as previously described (55). Briefly, neutrophils (1 × 10^6^) were stained with 0.5 μM CellTracker Red CMPTX dye (Invitrogen, Thermo Fisher Scientific) for 15 minutes at 37°C in rotation. Then, 5 × 10^4^ stained cells were resuspended in modified HBSS (20 mM HEPES at pH 7.4, 150 mM NaCl, 4 mM KCl, 1.2 mM MgCl_2_, and 10 mg/mL glucose) containing 10% heat-inactivated FBS and added to wells carved out on the agarose pad at the edges of a 35 mm glass-bottom dish with 20 mm microwell #1.5 cover glass (Cellvis). Cells were allowed to migrate toward the center well containing 100 nM WKYMVm (W3020, MilliporeSigma) for 1–4 hours in a 37°C/5% CO_2_ incubator. Endpoint images of both the front (toward WKYMVm) and back (away from WKYMVm) sides of the neutrophil-containing wells were captured using a Zeiss Axiovert microscope with 5× or 10× objective lens. Total numbers of neutrophils that left the well were manually counted. The distance traveled by neutrophils at the endpoint was determined using ImageJ (NIH). Time-lapse videos were captured using environment-controlled Zeiss Colibri fluorescence or Zeiss LSM 880 confocal microscopes with 10× or 20× objective lens. The automated TrackMate plugin in ImageJ was used to track migrating neutrophils, and the chemotaxis tool plugin was used to analyze the migration data. Analysis of all migration experiments was blinded.

### Neutrophil treatment with TGF-β and BALF.

Bone marrow neutrophils were isolated from naive mice as described above, plated at 200,000 cells per well in 96-well plates, and treated with the following: complete RPMI, complete RPMI + 2 ng/mL porcine TGF-1 (R&D Systems, Bio-Techne), and BALF from saline- or bleomycin-treated mice collected in complete RPMI. Cells were incubated with the described treatments for 4 hours at 37°C, after which intracellular killing assays were performed as described above.

### Lung macrophage isolation and phagocytosis and killing assays.

Total lung macrophages were quantified from collagenase-digested lung cells by differential staining and plated at 200,000 cells per well in black-bottom, 96-well plates. After a 1-hour incubation to allow macrophages to adhere, all nonadhered cells were washed from the plates. Macrophages were then treated with heat-killed FITC-conjugated MRSA (MOI = 300) for 2 hours to allow for update of the bacteria, after which extracellular fluorescence was quenched with trypan blue. Ex/em was measured at 485 nm/535 nm to measure intracellular fluorescence as a readout of phagocytosis of MRSA particles. Phagocytosis was normalized to macrophage survival as quantified via LDH assay. As a secondary assay for phagocytosis of nonopsonized MRSA, pHrodo Red *S*. *aureus* bioparticles (Invitrogen, Thermo Fisher Scientific) were used according to manufacturer instructions. Intracellular survival of MRSA as a readout of bacterial killing and phagocytosis of opsonized MRSA by macrophages were measured as described above in the neutrophil assay section.

### Statistics.

Statistical analyses were performed using GraphPad Prism software. For survival studies, a log-rank test was used to determine significance. For studies involving the comparison of 2 groups, an unpaired 2-tailed Student’s *t* test was used to compare normally distributed data, and a Mann-Whitney *U* test was used to compare data that were not normally distributed. For studies involving the comparison of more than 2 groups, a 1-way ANOVA followed by a Tukey posttest was used to compare normally distributed data, and a Kruskal-Wallis test followed by Dunn’s multiple-comparison posttest was used to compare data that were not normally distributed. Experiments were performed at least twice unless otherwise noted in figure legends. Experiment-specific statistical information is given in individual figure legends. A *P* value of less than 0.05 was considered significant; *P < 0.05, **P < 0.01, ***P < 0.001, and ****P < 0.0001.

### Study approval.

Animal experiments were performed in accordance with the University of Michigan’s IACUC-approved protocol (Protocol PRO00008731, “Immunobiology of the Lung in Fibrosis and Transplant”).

## Author contributions

HIWN designed and performed experiments, collected and analyzed data, and wrote and edited the manuscript. SJE performed experiments and analyzed data. SS performed, imaged, and analyzed under agarose chemotaxis experiments. CAP advised and assisted with planning of under agarose chemotaxis assays. XZ assisted with experiments and data analysis and edited the manuscript. DNO designed, performed, and analyzed data from survival experiments and edited the manuscript. BBM designed experiments, analyzed data, and edited the manuscript.

## Supplementary Material

Supplemental data

Supplemental table 2

Supplemental video 1

Supplemental video 2

Supplemental video 3

Supplemental video 4

Supplemental video 5

Supplemental video 6

## Version 1 01/06/2022

In-Press Preview

## Version 2 02/22/2022

Electronic publication
