# Supplementary material for: Fibrotic lung disease inhibits immune responses to staphylococcal pneumonia via impaired neutrophil and macrophage function
Source: JCI Insight. 2022 Feb 22;7(4):e152690. doi: 10.1172/jci.insight.152690 (PMC8876506; doi:10.1172/jci.insight.152690)
Supplement: Supplemental data [file jciinsight-7-152690-s027.pdf]

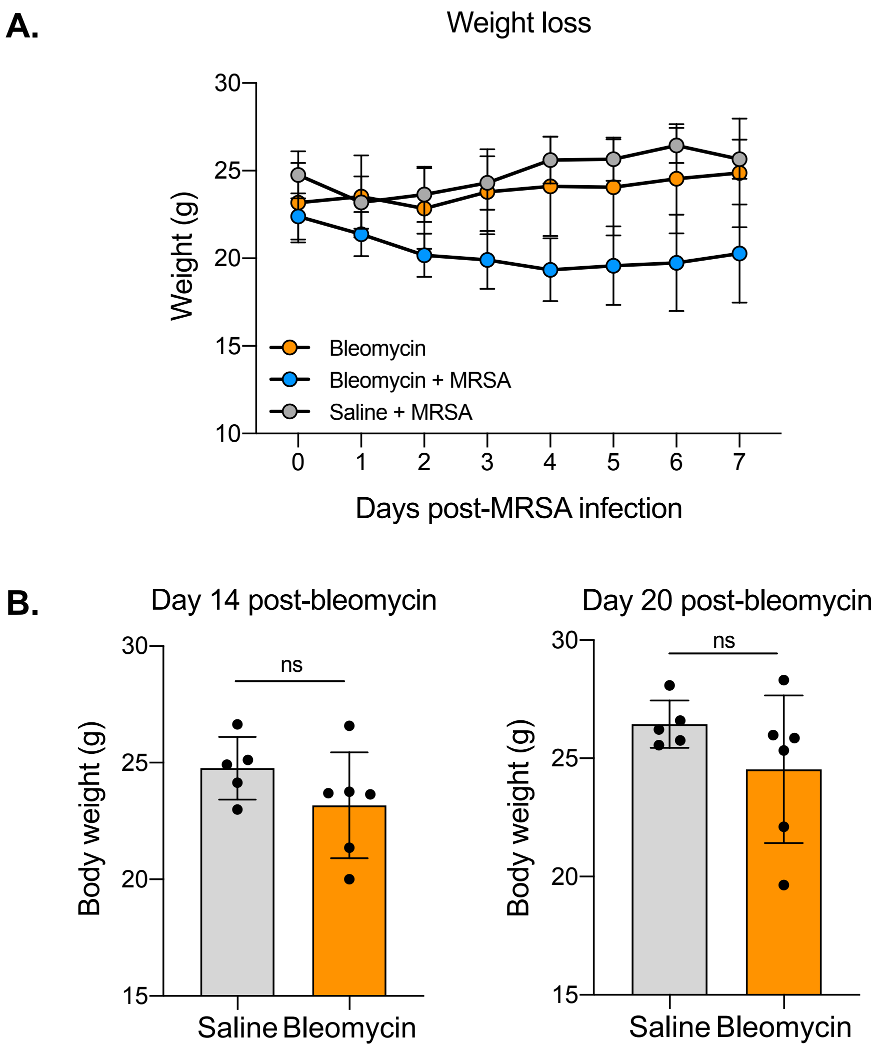

Supplemental Figure 1: Bleomycin does not significantly decrease mouse body weight 14 or 20 days after treatment. (A) Absolute body weight of mice treated with bleomycin or saline 14 days prior to infection with MRSA ($7x10^7$ CFU / mouse). **(B)** Direct comparison of absolute body weight of uninfected mice 14 and 20 days after treatment with saline or bleomycin (Day 0 and Day 6 in **A**, respectively). Data from one experiment. Bars represent the means +/- S.D. Statistical analysis by unpaired t-test.

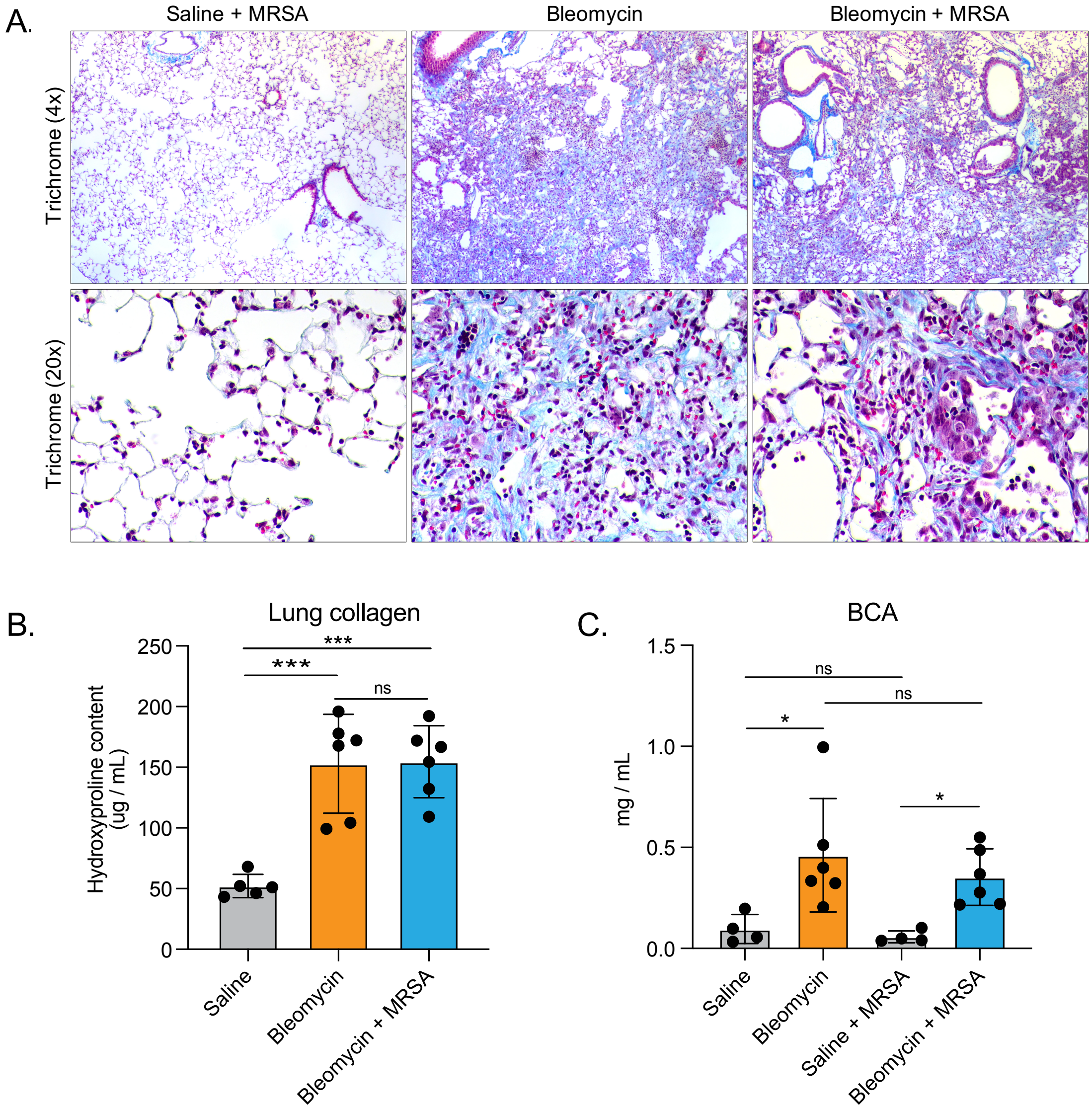

Supplemental Figure 2: Neither increased collagen deposition nor increased lung injury explain increased mortality after fibrotic lung injury and infection. (A) Lung sections from mice treated with saline + MRSA, bleomycin, or bleomycin + MRSA ($1x10^7$ CFU) and stained with Masson's trichrome. Scale bars represent 250 μm (top row) and 50 μm (bottom row). **(B)** Hydroxyproline assay from mice treated with saline (n = 5), bleomycin (n = 6), or bleomycin + MRSA (n = 6). Mice were infected with $7x10^7$ CFU MRSA 14 days after bleomycin or saline treatment and hydroxyproline was quantified 7 days post-infection. Representative of two independent experiments. Bars represent the means +/- S.D. Statistical analysis by one-way ANOVA with Tukey's multiple comparisons. *** = $p < 0.001$. **(C)** BCA assay to quantify total protein in BALF from mice treated with saline (n = 4), bleomycin (n = 6), saline + MRSA (n = 4), or bleomycin + MRSA (n = 6) ($1x10^7$ CFU), 21 days after bleomycin or saline treatment. Data are from a single experiment. Bars represent the means +/- S.D. Statistical analysis by Kruskal-Wallis test with Dunn's multiple comparisons. * = $p < 0.05$.

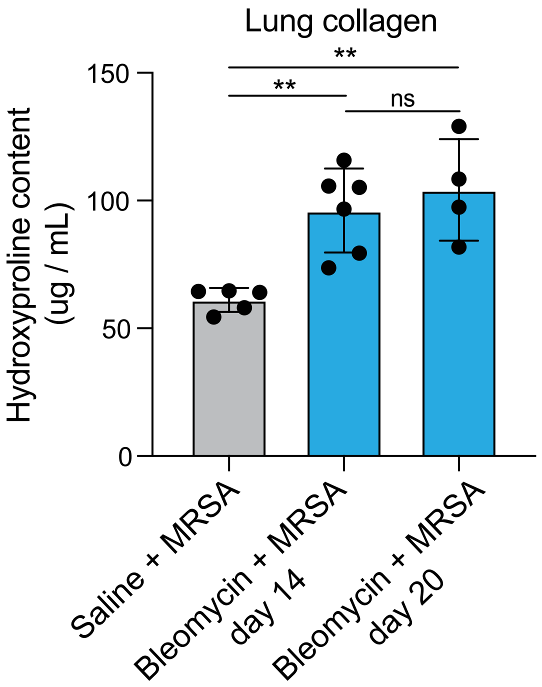

Supplemental Figure 3: Similar collagen levels 15 and 21 days post-bleomycin treatment. Hydroxyproline assay to measure lung collagen in mice were treated with saline (n = 5) or bleomycin (day 14 n = 6, day 20 n = 4) and then infected with $1x10^7$ CFU MRSA on either day 14 or day 20 post saline or bleomycin treatment. Lungs were harvested 24 hours post-infection. Data are from a single experiment. Bars represent the means +/- S.D. Statistical analysis by one-way ANOVA and Tukey's multiple comparisons. ** = $p < 0.01$.

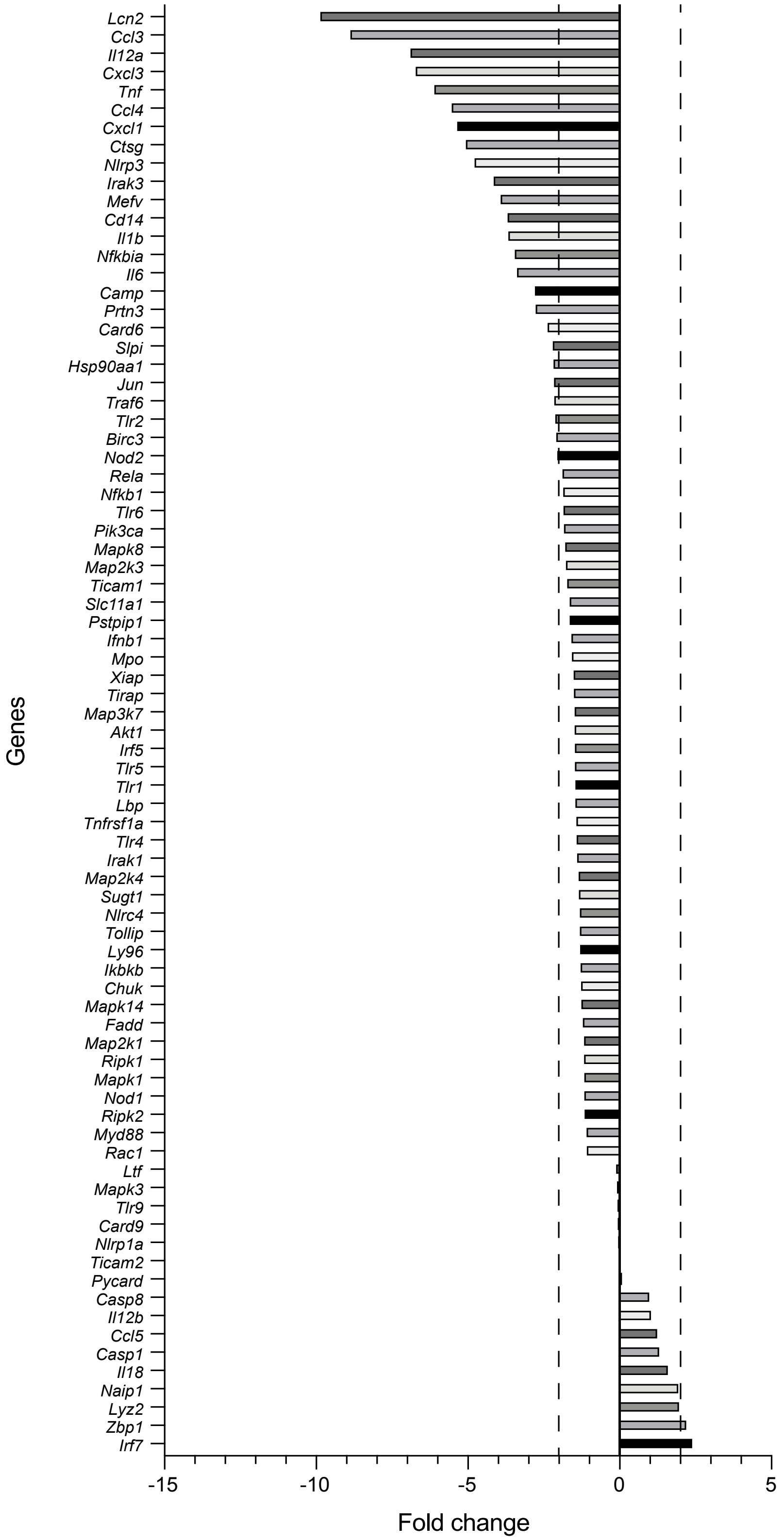

Supplemental Figure 4: Complete antibacterial response PCR array fold changes. Antibacterial response PCR array (QIAGEN) was used to measure gene expression of total lung cells from bleomycin + MRSA or saline + MRSA-treated mice ($1x10^7$ CFU). All measured genes shown as fold change of bleomycin + MRSA samples relative to saline + MRSA. Dashed lines designate threshold for differential expression (fold change +/- 2). Results are the average of two independent experiments. Samples from each experiment represent 4-5 combined biological replicates.

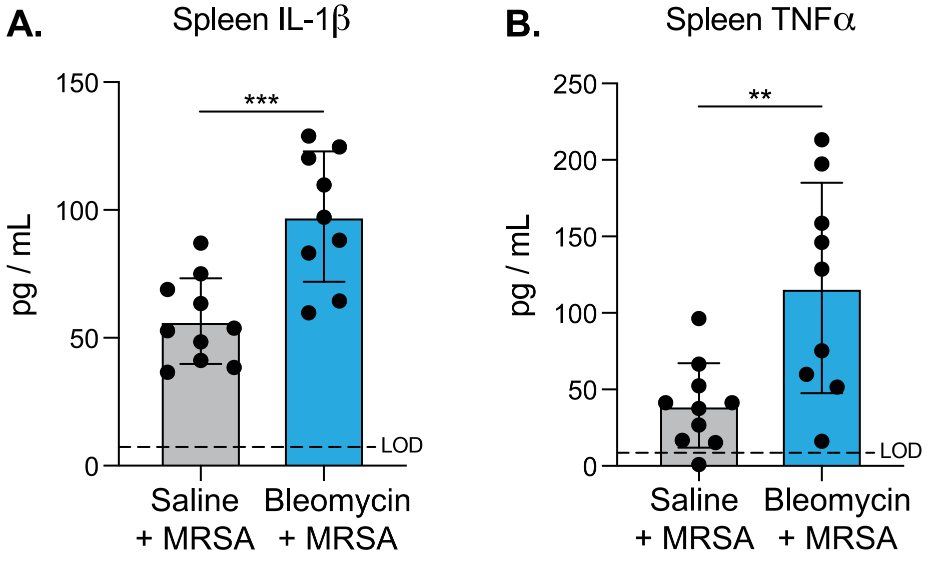

Supplemental Figure 5: Increased bacterial burden in spleen after fibrotic lung injury correlates with increased splenic proinflammatory cytokines. (A) Spleen IL-1β as measured via ELISA from mice treated with either saline + MRSA (n = 10) or bleomycin + MRSA (n = 9) ($1x10^7$ CFU). Data combined from two independent experiments. LOD = limit of detection. Bars represent the means +/- S.D. Statistical analysis by unpaired t-test. *** = $p < 0.001$. **(B)** Spleen TNFα as measured by ELISA from mice described in a. LOD = limit of detection. Bars represent the means +/- S.D. Statistical analysis by unpaired t-test. ** = $p < 0.01$.

Saline + MRSA

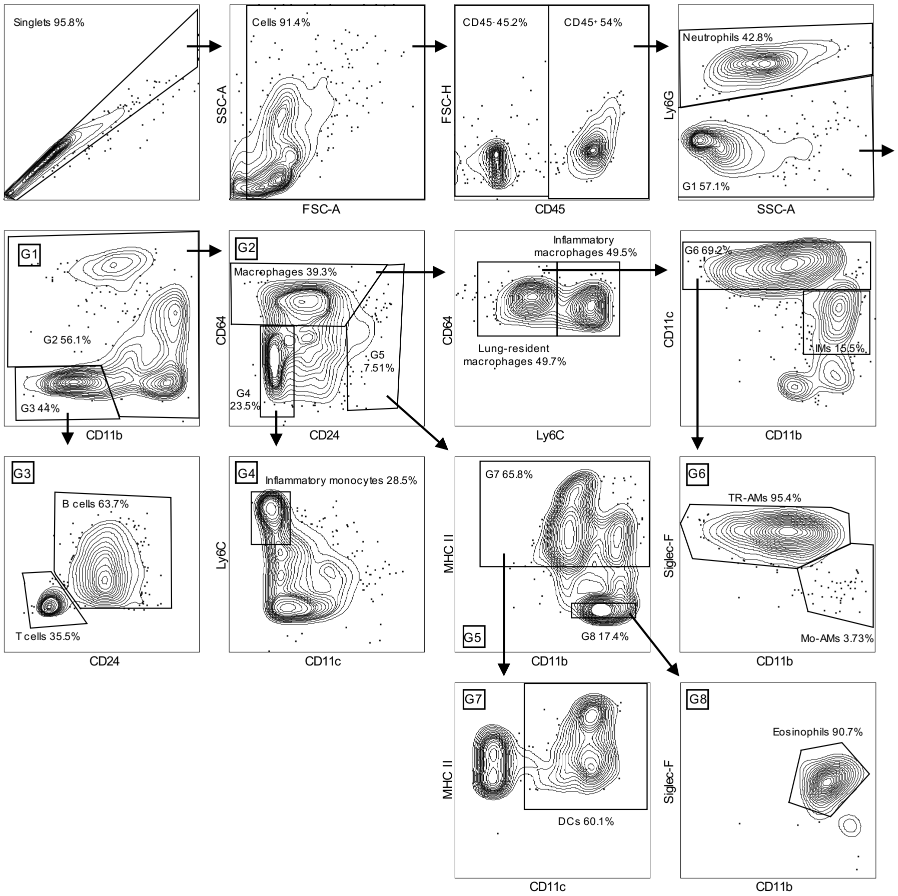

Bleomycin + MRSA

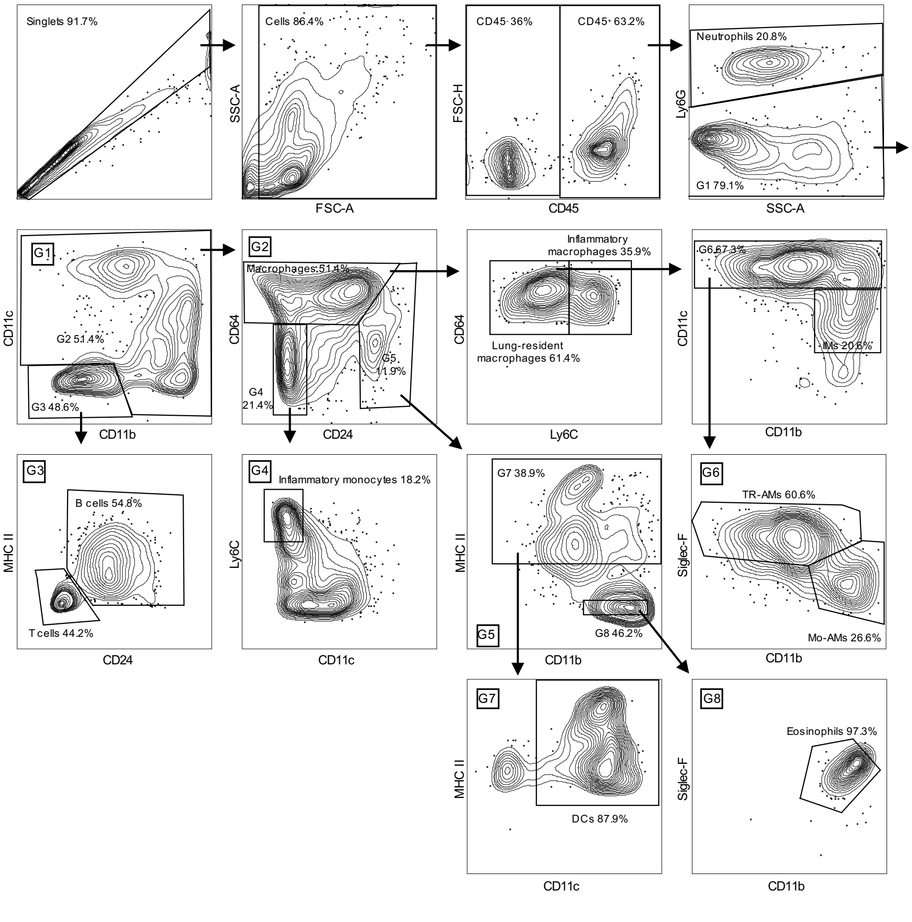

Supplemental Figure 6: Gating strategy for flow cytometric quantification of $CD45^+$ lung cells. Gating is shown from representative saline + MRSA and bleomycin + MRSA samples.

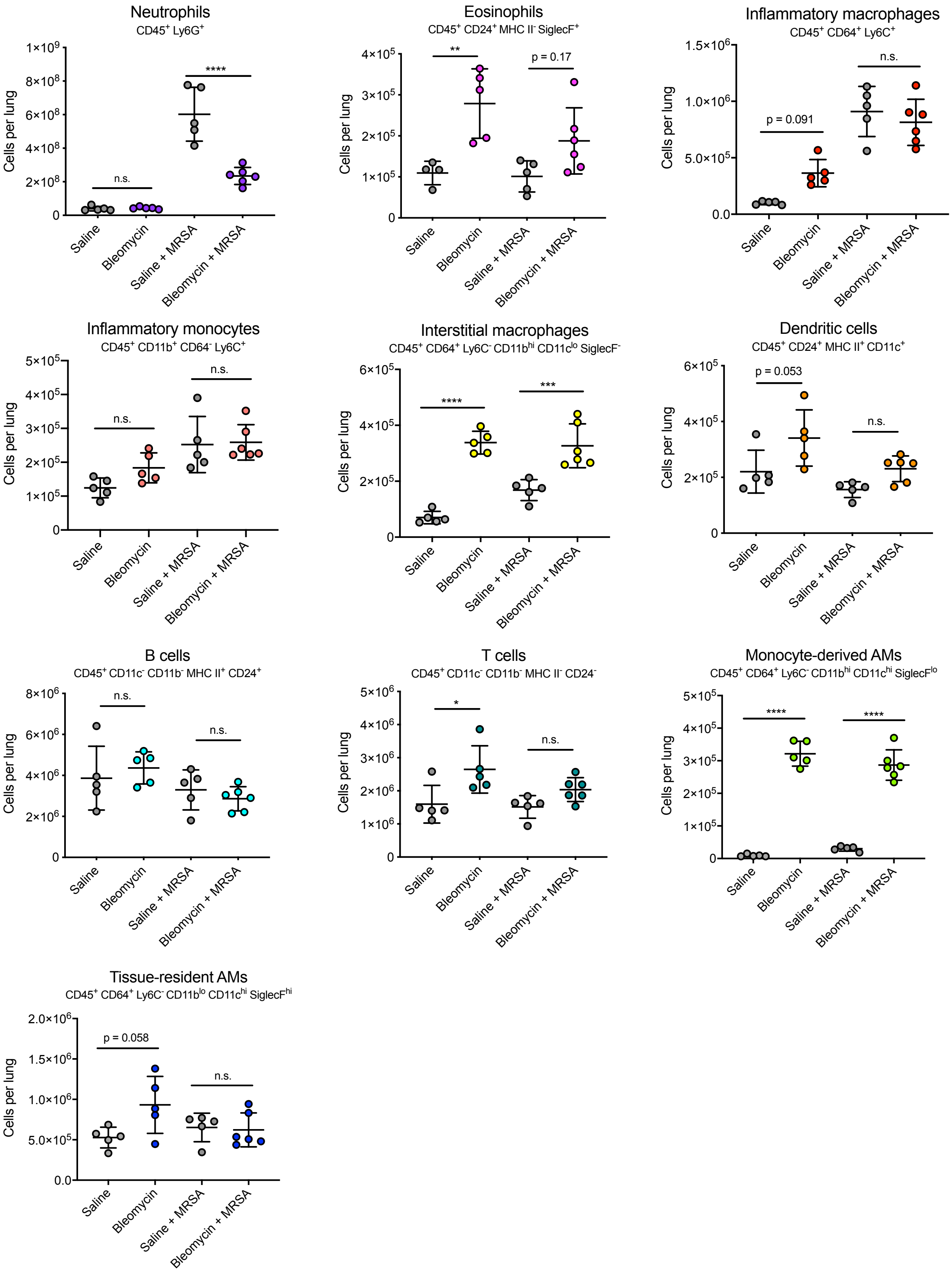

Supplemental Figure 7: Quantification of cell types isolated from saline and bleomycin-treated lungs after infection. Quantification of the numbers of indicated cell types isolated from saline (n = 5), bleomycin (n = 5), saline + MRSA (n = 5), and bleomycin + MRSA (n = 6)-treated mice (1x10^7 CFU). Representative of two independent experiments. Bars represent the means +/- S.D. Statistical analysis by one-way ANOVA with and Tukey's multiple comparisons. * = $p < 0.05$, ** = $p < 0.01$, *** = $p < 0.001$, **** = $p < 0.0001$

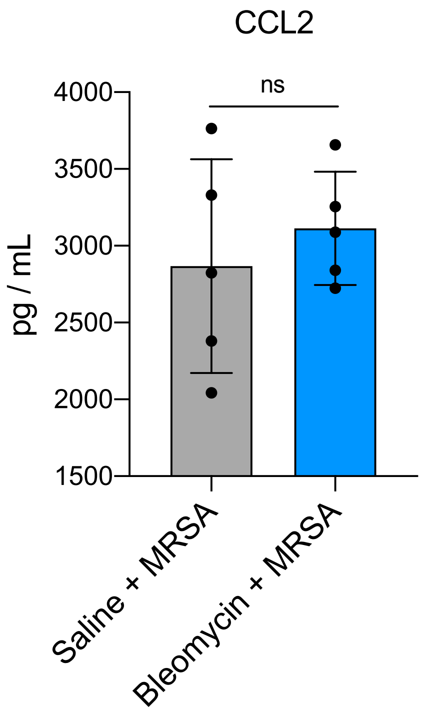

Supplemental Figure 8: No difference in lung CCL2 levels after infection. Lung CCL2 as measured via ELISA from mice treated with either saline + MRSA (n = 5) or bleomycin + MRSA (n = 5) ($1x10^7$ CFU). Data from one experiment. Bars represent the means +/- S.D. Statistical analysis by unpaired t-test.

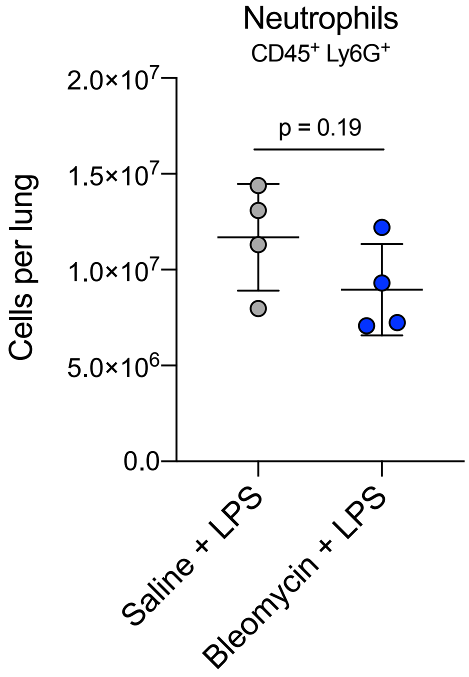

Supplemental Figure 9: No difference in neutrophil recruitment after LPS treatment. Quantification of lung neutrophils identified via flow cytometry from mice treated with saline + LPS (n = 4) and bleomycin + LPS (n = 4). 25 ug per mouse of *Pseudomonas aeruginosa* LPS was administered 20 days after bleomycin or saline and lungs were harvested 24 days after LPS treatment. Data from one experiment. Bars represent the means +/- S.D. Statistical analysis by unpaired t-test.

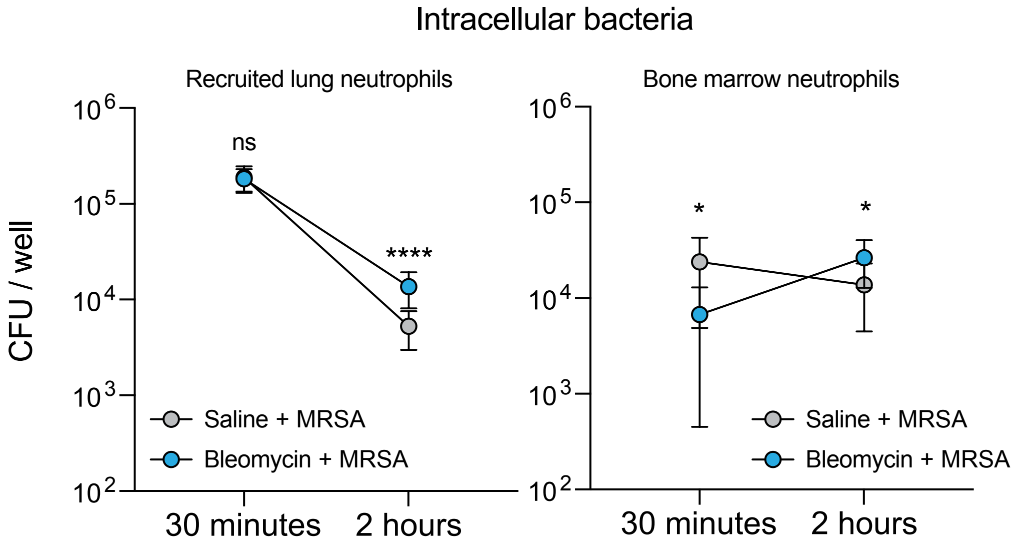

Supplemental Figure 10: Defects in intracellular killing of MRSA by lung and bone marrow neutrophils. Intracellular MRSA CFU quantified 30 minutes and 2 hours after ex vivo infection of lung and bone marrow neutrophils as described in Figure 5C. Neutrophils were isolated 21 days after saline or bleomycin treatment. Representative of two independent experiments. Bars represent the means +/- S.D. Statistical analysis by unpaired t-test. * = $p < 0.05$, **** = $p < 0.0001$

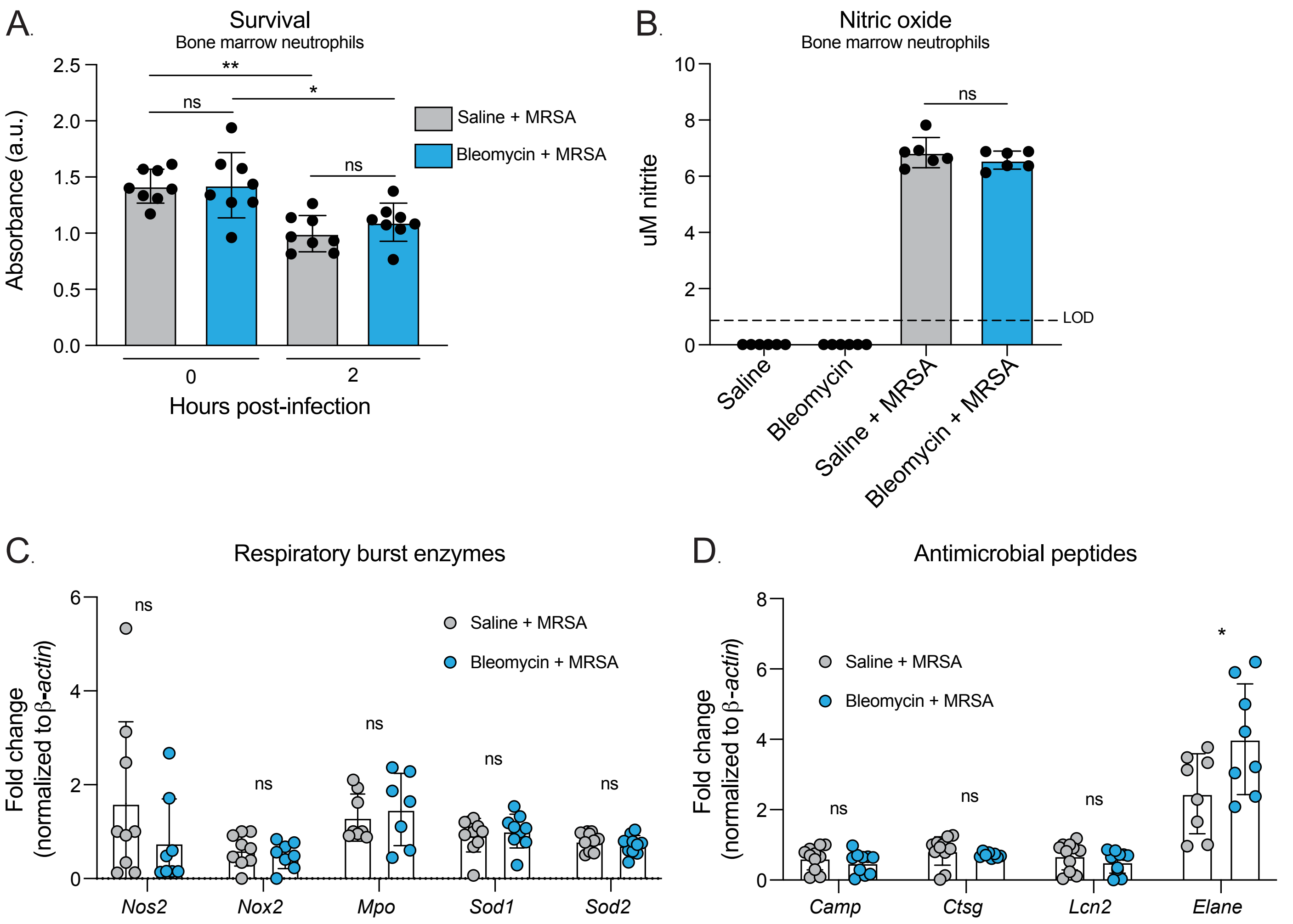

Supplemental Figure 11: Neutrophil survival, nitric oxide production, and expression of free radical-generating enzymes and antimicrobial peptides is not affected by fibrotic lung injury. (A) Survival of bone marrow neutrophils after ex vivo MRSA infection. Neutrophils isolated from saline or bleomycin-treated mice (2-3 mice per group). Representative of two independent experiments, dots represent technical replicates of pooled cells. Bars represent the means +/- S.D. Statistical analysis by one-way ANOVA with Tukey's multiple comparisons. * = $p < 0.05$, ** = $p < 0.01$. **(B)** Nitric oxide production by bone marrow neutrophils after ex vivo MRSA infection. Cells isolated from saline or bleomycin-treated mice (2-3 mice per group). Data is from one experiment; dots represent technical replicates of pooled cells. LOD = limit of detection. Bars represent the means +/- S.D. Statistical analysis by ANOVA with Tukey's multiple comparisons. **(C)** *Nos2*, *Nox2*, *Mpo*, *Sod1*, and *Sod2* expression by bone marrow neutrophils after ex vivo infection with MRSA. Cells isolated from saline or bleomycin -treated mice. Data from two combined independent experiments, dots represent technical replicates of pooled cells from 4-5 mice per group. Bars represent the means +/- S.D. Statistical analysis by unpaired t tests. **(D)** *Camp*, *Lcn2*, *Ctsg*, and *Elane* expression by bone marrow neutrophils after ex vivo infection with MRSA. Cells isolated from saline or bleomycin -treated mice. Data from two combined independent experiments, dots represent technical replicates of pooled cells from 4-5 mice per group. Bars represent the means +/- S.D. Statistical analysis by unpaired t tests.

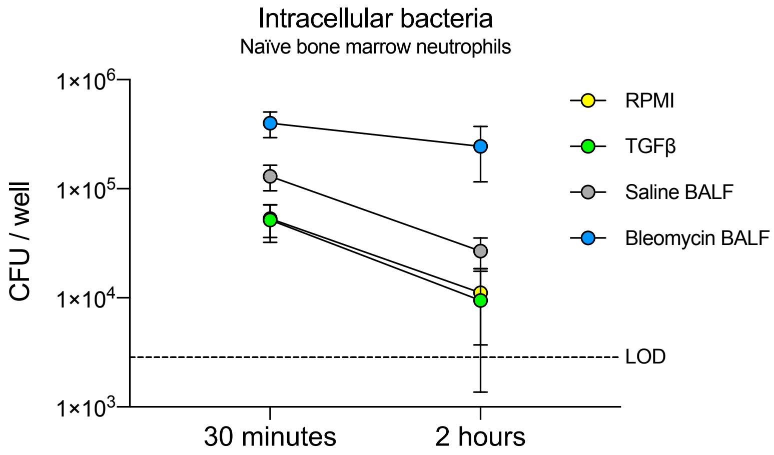

Supplemental Figure 12: Treatment with bleomycin BALF impairs intracellular MRSA killing by naïve neutrophils. Intracellular MRSA CFU quantified 30 minutes and 2 hours after ex vivo infection of bone marrow neutrophils as described in Figure 6A. Neutrophils were treated for 4 hours with complete RPMI, complete RPMI + TGFβ (2 ng/mL), or BALF from saline or bleomycin-treated mice collected in complete RPMI. Representative of two independent experiments. Bars represent the means +/- S.D.

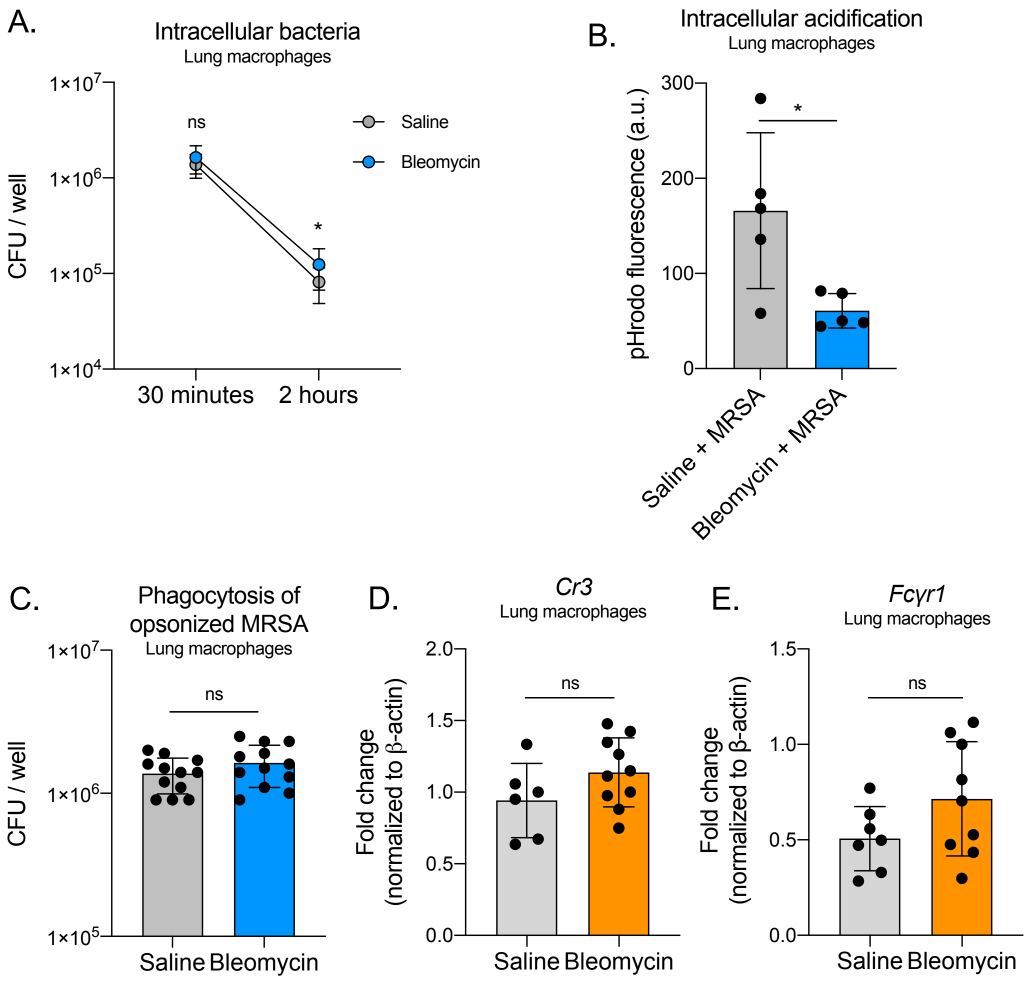

Supplemental Figure 13: Macrophage phagocytosis of non-opsonized MRSA, but not opsonized MRSA, is impaired in fibrosis. (A) Intracellular MRSA CFU quantified 30 minutes and 2 hours after ex vivo infection of lung macrophages as described in Figure 7B. Macrophages were isolated 21 days after saline or bleomycin treatment. Representative of two independent experiments. Bars represent the means +/- S.D. Statistical analysis by unpaired t-test. * = $p < 0.05$. **(B)** Lung macrophage phagocytosis as measured by pH-sensitive *S. aureus* bioparticles. Following uptake of bioparticles and acidification of the macrophage phagolysosome, fluorescent signal is detectable. Lung macrophages were isolated 21 days after saline or bleomycin treatment and treated with bioparticles ex vivo for 2 hours. Data from one experiment, dots represent technical replicates of pooled cells (n = 5). Bars represent the means +/- S.D. Statistical analysis by unpaired t-test. * = $p < 0.05$. **(C)** Phagocytosis of opsonized MRSA after 30 minutes by lung macrophages infected ex vivo. Representative of two independent experiments. Bars represent the means +/- S.D. Statistical analysis by unpaired t-test. **(D-E)** *Cr3* (D) and *Fcγr1* (E) expression by uninfected lung macrophages isolated from mice 21 days after saline or bleomycin treatment. Dots represent technical replicates of pooled cells (n = 6-10). Cells from 2-3 mice per group. Data from two combined experiments. Bars represent the means +/- S.D. Statistical analysis by unpaired t-test.

Supplemental Videos 1-4: Under agarose chemotaxis assay measuring bone marrow neutrophil migration towards formyl peptide receptor agonist WKYMVm1 (100 nM) for 3 hours with 20 second time intervals using Zeiss Colibri fluorescence microscope at 10x objective. Videos 1 and 2 show neutrophils from saline (Video 1) or bleomycin treated (Video 2) mice migrating from the front side of the wells towards the WKYMVm1 gradient. Videos 3 and 4 show migration from the back side of the wells by neutrophils from saline (Video 3) or bleomycin treated (Video 4) mice, indicating a chemotactic response.

Supplemental Videos 5-6: Bone marrow neutrophils from saline (Video 5) or bleomycin-treated (Video 6) mice were allowed to migrate under agarose toward formyl peptide receptor agonist WKYMVm (100 nM) for 1.5 hours with 18 sec time intervals using Zeiss LSM880 confocal microscope at 20x objective. Migrated cells were tracked with TrackMate plugin in ImageJ. Different parameters of cell migration were determined by analyzing the migration data with the chemotaxis tool and applying threshold distance of > 50 µm. Quantification of migration parameters from two averaged positions for each treatment is shown in Supplementary Table 1.

Supplemental Table 1. Quantification of neutrophil migration.

	Directionality	Accumulated distance	Euclidean distance	Speed (µm/min)
**Saline**	0.6	254	132	19
**Bleomycin**	0.4	191	69	14
